# Viruses Infecting Trees and Herbs That Produce Edible Fleshy Fruits with a Prominent Value in the Global Market: An Evolutionary Perspective

**DOI:** 10.3390/plants11020203

**Published:** 2022-01-13

**Authors:** Lizette Liliana Rodríguez-Verástegui, Candy Yuriria Ramírez-Zavaleta, María Fernanda Capilla-Hernández, Josefat Gregorio-Jorge

**Affiliations:** 1 Departamento de Biotecnología, Universidad Autónoma Metropolitana, Ciudad de Mexico 09340, Mexico; lizette.liliana@gmail.com; 2 Cuerpo Académico Procesos Biotecnológicos, Universidad Politécnica de Tlaxcala, Av. Universidad Politécnica 1, San Pedro Xalcaltzinco 90180, Mexico; candy.y.ramirez@gmail.com (C.Y.R.-Z.); fernandacapilla1@gmail.com (M.F.C.-H.); 3 Consejo Nacional de Ciencia y Tecnología, Universidad Politécnica de Tlaxcala, Av. Insurgentes Sur 1582, Col. Crédito Constructor, Ciudad de Mexico 03940, Mexico

**Keywords:** crop, fruit, pathogen, disease, plants, eudicots, monocots, magnoliids, yield, diagnosis, virus

## Abstract

Trees and herbs that produce fruits represent the most valuable agricultural food commodities in the world. However, the yield of these crops is not fully achieved due to biotic factors such as bacteria, fungi, and viruses. Viruses are capable of causing alterations in plant growth and development, thereby impacting the yield of their hosts significantly. In this work, we first compiled the world′s most comprehensive list of known edible fruits that fits our definition. Then, plant viruses infecting those trees and herbs that produce fruits with commercial importance in the global market were identified. The identified plant viruses belong to 30 families, most of them containing single-stranded RNA genomes. Importantly, we show the overall picture of the host range for some virus families following an evolutionary approach. Further, the current knowledge about plant-virus interactions, focusing on the main disorders they cause, as well as yield losses, is summarized. Additionally, since accurate diagnosis methods are of pivotal importance for viral diseases control, the current and emerging technologies for the detection of these plant pathogens are described. Finally, the most promising strategies employed to control viral diseases in the field are presented, focusing on solutions that are long-lasting.

## 1. Introduction

Since the appearance of agriculture, far earlier than previously thought, a few places around the world were sites of origin for the modern crops we see today [1,2,3]. In a process that took thousands of years, cultivated plants suffered the displacement of wild characteristics and enrichment of suitable traits such as yield, disease resistance, abiotic stress tolerance, and/or quality [4,5]. Among the total plant species on earth, around 10% of them have a documented use, from which ~5000 plant species are a source for human food [6,7,8,9]. In the case of fruit trees and herbs, edible fruits were harvested from the wild and constituted the earliest source of food for humans [10,11]. Nowadays, more than 2000 species are used as food in the tropics, but only a fraction of them have been domesticated, and a very tiny number are of significant commercial importance in the global market [12].

Although everyone is familiar with the traits of fruits, such as increased size of the pericarp tissue and high sugar concentrations, these traits evolved initially for dispersing seeds by means of megafaunal species [11,12,13,14]. Therefore, Spengler argues that fleshy fruits were evolutionary adaptations that facilitated a stronger mutualistic bond between plants and humans during domestication of the former [15]. In any case, a wide range of fruits is consumed around the world; bananas, watermelons, apples, oranges, and grapes, to mention some of them, are the most popular [16,17]. More importantly, fruits are known as amazing natural medicines, preventing many chronic diseases [18,19,20]. Therefore, WHO and FAO have made efforts to promote fruit and vegetable consumption around the world [21].

Putting aside the shortage of fruits and, consequently, higher prices caused by the COVID-19 pandemic, the biggest challenge faced by fruit crops has always been the range of pathogens that can cause disease [22,23,24]. Among the plant pathogens, which include fungi, bacteria, and viruses, the major group causing reduction of fruit quality, premature fruit drop, and yield loss is viruses [25,26]. Harmful plant virus diseases are of considerable concern for humanity since they are capable of decreasing food supplies and, therefore, threatening food security. The situation is becoming worse because the human population is growing, soil fertility is declining, and global warming is changing the weather patterns [27,28,29,30].

## 2. Edible Fruits of the World: Which of Them Feed the World?

Although technically nuts and grains are also fruits, we have restricted our review to trees or herbs that produce fleshy seed-associated structures that typically are sweet or sour and edible when raw [31]. Taking into account that definition of fruit, an extensive search by combining multiple data sources allowed us to identify those trees and herbs that produce edible fleshy fruits (EFFs) [8,11,12,32,33,34,35,36,37,38,39,40]. Accordingly, more than 2000 plant species that produce EFFs were identified (Supplementary Table S1). These EFFs were classified into 140 plant families and 47 orders. It is worth mentioning that some EFFs are borderline because their fleshy tissue is in a limited amount; however, they were included since their consumption has been reported to some extent. The classification of EFFs into higher ranks showed that they mostly belong to eudicots (2119), followed by monocots (74) and magnoliids (42) (Table 1).

EEFs classified as eudicots corresponded to 438 genera, 119 families, and 38 orders, whereas monocots and magnoliids were classified into 32, 16, and 7 and 15, 5, and 2, respectively (Supplementary Figure S1). Until recently, the relationship between magnoliids, monocots, and eudicots had not been conclusively resolved [41]. For instance, members of magnoliids are dicotyledonous plants that retain some primitive anatomic and morphological characteristics when compared to eudicots and monocots [42]. Therefore, from an evolutionary point of view, magnoliids correspond to early-diverging angiosperms, followed by monocots and eudicots [41,43,44].

**Figure 1:**
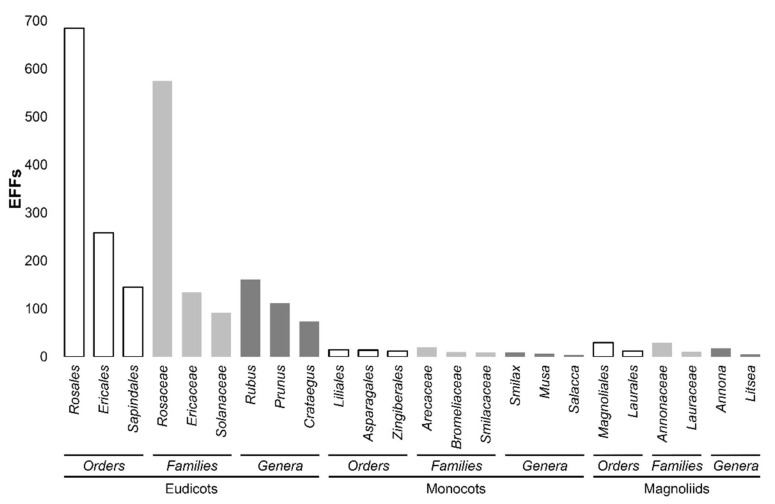
Taxonomy of all EFFs in the world. Classification of more than 2000 EFFs shows the most representative orders, families, and genera to which they belong. Note the increasing inclusivity of the higher taxonomic ranks such as families, orders, and finally clades (eudicots, monocots, and magnoliids). Complete information is found in Supplementary Table S1.

Figure 1 shows the genera, families, and orders to which EFFs mostly belong within the three clades. As observed, the vast amount of EFF species correspond to eudicots; specifically, plant species of the genera *Rubus* (161), *Prunus* (112) and *Crataegus* (74), constituted the largest family of *Rosaceae* (575), order Rosales (685) (Figure 1). EFFs belonging to families *Ericaceae* (135) and *Solanaceae* (92) were also numerous, in which the genera *Vaccinium* (56) and *Solanum* (36) were highly represented, respectively (Figure 1; Supplementary Table S1). In fact, numerous EFFs within the family *Rosaceae* are well known (cultivated), including apples (*Malus* sp.), pears (*Pyrus* sp.), plums, and cherries (*Prunus* sp.), among others. However, many more EFFs within the family *Rosaceae* are found in the wild such as Ghingaroo (*Pyracantha crenulata*), Mehul (*Pyrus pashia*), and *Rubus niveus*. In the case of EFFs classified within the clades of monocots and magnoliids (Figure 1), the same situation is true; namely, some of them have been domesticated (i.e., *Musa acuminata* and *Annona muricata*), whereas others remain in the wild (i.e., *Musa dasycarpa* and *Annona glabra*, respectively) (Supplementary Table S1).

**Figure 2:**
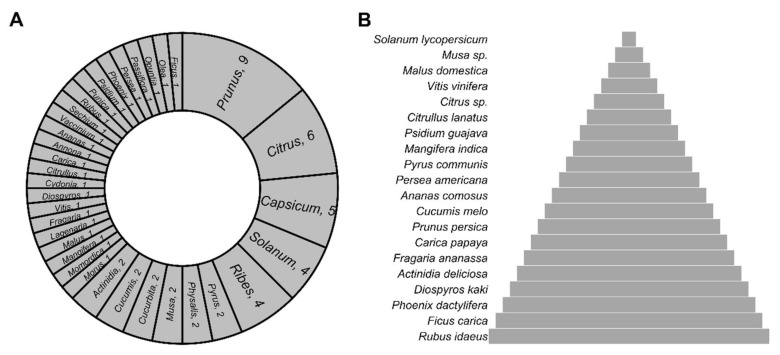
Commercially important EFFs. (**A**) Genera in which the 64 EFFs with a prominent role in the global market belong. The number of species in each genus is indicated. (**B**) The top 20 most important EFFs are shown as a pyramid, which is divided into layers. The top layer represents the most traded EFF and the bottom layer the less traded, respectively.

Despite the wide range of options for EFFs, only a tiny fraction of them is consumed by the world [41,42,43,44,45,46,47]. For example, although tomatoes and bananas do not grow in every corner of the world, they are ubiquitous. This is due to advances in transportation, agreements and consumer preferences. In this regard, we have identified 64 species of EFFs that constitute valuable products in the international marketplace (Supplementary Table S2). Out of the 64 EFFs, 58 correspond to eudicots, whereas only four and two correspond to monocots and magnoliids, respectively (Supplementary Table S2). Such commercially important EFFs are classified into 35 genera, several species being conspicuous that belong to *Prunus*, *Citrus*, *Capsicum*, *Ribes*, and *Solanum*, mainly (Figure 2A). Specifically, 20 out of the 64 EFFs have a prominent value in the world market; tomato (*Solanum lycopersicum*), banana (*M. acuminata*), apple (*Malus domestica*), and grapes (*Vitis vinifera*) standing out as the most traded EFFs (Figure 2B) [16,17].

Although there is a recommendation of consuming at least 400 g of fruits and vegetables daily [21], insufficient fruit intake is observed worldwide. On the other hand, fruits have diversity in their consumption because there are factors influencing consumer behavior and preferences. Some people prefer strawberries or grapes, whereas others like oranges, cherries, watermelons, or pineapples. Additionally, depending on availability, regional climate, and other factors, fruit production and consumption vary greatly from one country to the next, but on a global scale, there is one fruit that stands above all the rest: tomato (Table 2). In Table 2, besides showing the global production of the most popular EFFs in the world, it comes along with their market worth. As observed, tomatoes, bananas, watermelons, and apples are among the most consumed fruits in the world (Table 2).

The economic importance of EFFs is reflected by the fastest growth rates of exportation in recent years [52]. Although this is encouraging, the genetic potential of crops yield is constrained by abiotic and biotic factors, reaching at best only 20 percent of the full potential [53,54]. Moreover, out of the actual total capacity for annual food production, up to 40 percent is lost due to biotic factors, which are often more severe in developing countries than in developed countries [23,55]. Such declines in yield caused by plant pathogens are projected to increase under higher temperatures because of global warming. Therefore, EFF production continues to be the main challenge in tropical and semi-tropical areas around the world, particularly those expenses related to disease prevention and control.

## 3. A Co-Evolutionary Arms′ Race between Plants and Viruses: Major Families of Plant Viruses Affecting EFF Crops

Among the plant pathogens, viruses (and viroids), which are transmitted by a living organism called a vector, are the major infectious agents that cause plant disease [56,57,58]. Once a plant virus infects a susceptible host, it can spread to another plant by means of vector-mediated transmission (horizontally) or from parents to offspring (vertically) [59]. Although the aforementioned modes of transmission are the pathways by which most plant viruses spread in nature, other modes of transmission should be considered, such as wounds or abrasions in the plant surface caused by wind, contaminated soil, and water, as well as chewing by herbivores [60,61].

Plant systemic infection starts from the initial areas of virus inoculation, from which virions move through the plasmodesmata of plant cells until they reach the vascular bundles, resulting in the long-distance transport of the viral particles [61,62,63,64]. At each step of this process, there must be a basic level of molecular communication between the virus and its host [64,65]. Thus, the outcome of plant–virus interactions depends on the effectiveness of plant defense mechanisms and on the ability of the virus to counteract these host defense responses. Under this scenario, for millions of years, plants have evolved a series of mechanisms to cope with the invading virus; the first as based on the detection of pathogen-associated molecular patterns (PAMPs) that activates PAMP-triggered immunity (PTI) [66]. PTI, which is a non-specific response to a broad range of pathogens, may occasionally result in a hypersensitive response (HR), thereby restricting the proliferation of the virus. However, some viruses have circumvented this response by developing effector proteins encoded by avirulence (*Avr*) genes that interfere or suppress PTI [67]. In response, plants have evolved intracellular nucleotide-binding leucine-rich repeat (NLR) receptors, which recognize the Avr effectors in a highly specific manner to trigger the second mechanism of defense called effector-triggered immunity (ETI) [68]. Thus, the so-called co-evolutionary “arms race” between plants and viruses results in transcriptional and biochemical changes of the host either lead to a proper plant defense response (incompatible) or its colonization (compatible) [69,70,71,72]. In that sense, compatible plant–virus interactions may or may not show the main symptoms of viral diseases, including growth suppression, discoloration, deformations, necrosis, and impaired reproduction. These visible symptoms appear as a result of changes in starch metabolism, nitrogen metabolism, hormone metabolism, and water content, among others [73,74,75,76,77,78,79,80,81].

As described above, there is a cascade of mutual and complex interactions between the plant host and the virus [80,81]. Most viruses infecting plants are composed of RNA genomes, whereas a minority have DNA genomes [58]. Plant viruses can be further classified as positive-sense single-stranded RNA (+ssRNA), negative-sense single-stranded RNA (−ssRNA), single-stranded RNA viruses that Reverse Transcribe (ssRNA-RT), single-stranded DNA (ssDNA), double-stranded RNA (dsRNA), and double-stranded DNA viruses that Reverse Transcribe (dsDNA-RT) [82,83,84,85]. According to the International Committee for Taxonomy of Viruses (ICTV), classification is as follows: orders, families, subfamilies, genera, and species [84,85]. Currently, 1744 species of plant viruses are listed in the ICTV (2021) report, belonging to 31 families [84,85,86].

A decade ago, Scholthof and co-workers published the top ten viruses based on their scientific/economic importance [87]. However, Rybicki questioned their approach since it did not accurately reflect the most economically important viral crop pathogens [88]. More recently, Jones and Naidu revisited the global impact of virus diseases, noting that members of the begomoviruses, tospoviruses, and potyviruses endanger food security by causing devastating diseases in tropical and subtropical food crops [25]. Some of these crops belong to EFFs, such as tomato (*S. lycopersicum*), watermelon (*Citrullus lanatus*), zucchini (*Cucurbita pepo*), and cucumber (*Cucumis sativus*) [25]; however, a comprehensive picture of all viruses infecting trees or herbs that produce EFFs with economic importance in the world is scarce or lacking [87,88,89,90,91,92].

**Figure 3:**
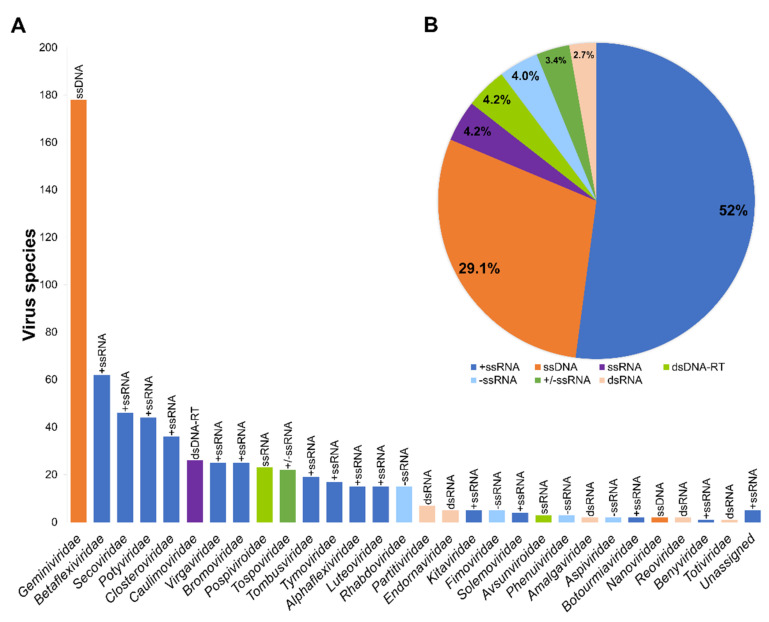
Classification of 617 plant viruses infecting EFFs. (**A**) The number of virus species identified in each family is shown. Virus families are indicated by color code according to the nature of their genomes (above each bar). (**B**) Percentage of viruses containing RNA (+ssRNA, −ssRNA, +/−ssRNA, ssRNA, and dsRNA) or DNA (ssDNA and dsDNA-RT) genomes.

Here, we have identified all viruses that infect trees and herbs that produce EFFs with an important role in the international marketplace. Rather than taking into account the total number of virus records (~1500), we focused on identifying unique virus species infecting the 64 EFFs (Supplementary Table S3). In that sense, 617 virus species were identified, which belong to 89 genera and 30 virus families (Figure 3A; Supplementary Table S3). According to the number of virus species identified, the most prominent families infecting EFFs were *Geminiviridae* (178), followed by *Betaflexiviridae* (62), *Secoviridae* (46), *Potyviridae* (44), and *Closteroviridae* (36) (Figure 3A; Supplementary Figure S2). On the other hand, taking into account the nature of their genomes, the classification of the 617 viruses showed that slightly more than half of them possess +ssRNA genomes (Figure 3B). Such viruses with +ssRNA genomes belong to 14 families: *Betaflexiviridae*, *Secoviridae*, *Potyviridae*, *Closteroviridae*, *Virgaviridae*, *Bromoviridae*, *Tombusviridae*, *Tymoviridae*, *Alphaflexiviridae*, *Luteoviridae*, *Kitaviridae*, *Solemoviridae*, *Botournaviridae*, and *Benyaviridae* (Figure 3A). Additionally, viruses with ssDNA genomes were found in a significant proportion, represented by members belonging to the families *Geminiviridae* and *Nanoviridae* (Figure 3B). Other forms of nucleic acid genomes were represented in minor proportions; viroids with ssRNA genomes constituted a significant proportion among this group (Figure 3B).

**Figure 4:**
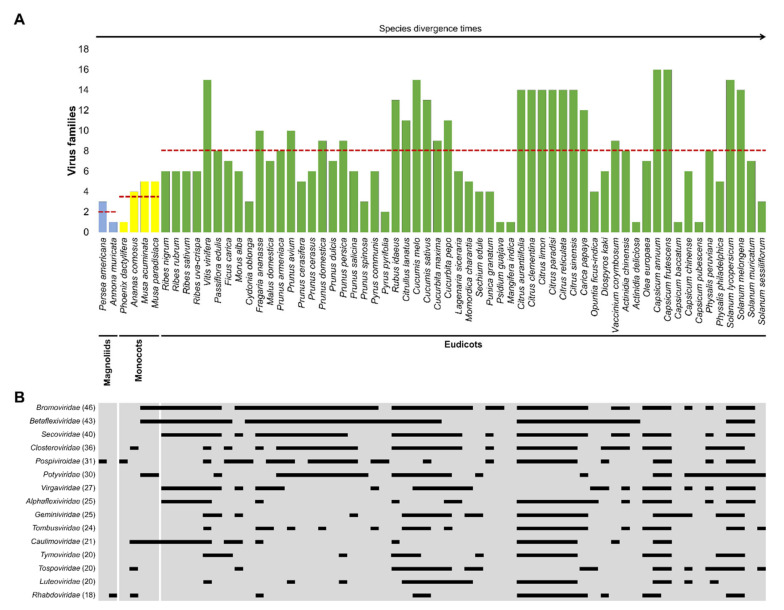
Virus families infecting the 64 commercially important EFFs. (**A**) Classification of EFFs according to plant divergence times [93], and references therein], as well as the number of virus families infecting them. The average number of virus families infecting EFFs classified into magnoliids, monocots, and eudicots is indicated with a red dashed line. (**B**) The host range for the first 15 virus families is exemplified with thin rectangles (filled). Complete information is found in Supplementary Table S4.

Even though 80 percent of the identified viruses infecting EFFs corresponded to either +ssRNA or ssDNA genomes (Figure 3B), these proportions did not correlate necessarily with their prevalence among the 64 EFFs. A clear example was the family *Geminiviridae*, in which 128 out of 174 virus species were identified in a single plant species, *S. lycopersicum*. This encouraged us to examine the host range of virus families among the 64 commercially important EFFs. The approach followed was according to an evolutionary perspective, namely, taking into account the divergence times of EFFs [41,43,44,93]. Thus, we organized the 64 economically important EFFs as follows: magnoliids first, then monocots, and finally eudicots (Supplementary Table S4). Within the magnoliids clade, for example, *Persea americana* (avocado) and *Annona muricata* (soursop) belong to *Lauraceae* (order Laurales) and *Annonaceae* (order Magnoliales) families, whereas monocot species such as *Phoenix dactylifera*, *Ananas comosus*, and *Musa* sp. belong to *Arecaceae* (order Arecales), *Bromeliaceae* (order Poales) and *Musaceae* (order Zingiberales) families. In the case of eudicot species (58), these EFFs were classified into 17 families and 12 orders (Supplementary Table S4). All this information is summarized in Figure 4, with an attempt to offer a full picture of virus families infecting the 64 EFF species. As shown in Figure 4A, even though the number of virus families infecting each of the 64 EFF species differed in general, it was conspicuous that eudicot species contained the highest number of virus families compared to monocots and magnoliids (Figure 4A). Specifically, EFFs belonging to eudicots showed to be infected by eight virus families on average, whereas monocots and magnoliids were infected by 3.75 and 2 virus families, respectively (Figure 4A). Eudicot species such as tomato (*S. lycopersicum*), pepper (*Capsicum annuum*), and grape (*V. vinifera*) were found to be infected by the highest number of plant viruses, whereas mango (*Mangifera indica*) and guava (*Psidium guajava*) were found to be infected by a single virus species. Specifically, in the cases of *S. lycopersicum* and *C. annuum*, 207 and 118 different virus species were identified, respectively (Supplementary Table S4).

The higher number of virus families found among EFFs belonging to eudicots could be the result of host range expansion during plant evolution, namely, an increase in the number of potential hosts or also by host shifts. Therefore, the overall host range distribution of major virus families infecting the 64 EFF species was performed (Supplementary Table S4). As observed in Figure 4B, the host range distribution of 15 out of 30 virus families, encompassing 92.4% of all viruses found in this study, is extensive among eudicots. Families such as *Bromoviridae, Betaflexiviridae*, *Secoviridae*, and *Closteroviridae* showed the widest host range distribution (Figure 4B). The numbers of plant species infected by these families were 46, 43, 40, and 36, respectively (Supplementary Figure S3). Examples of virus species representative of virus families with a wide range of hosts within eudicot EFFs are cucumber mosaic virus (CMV; *Cucumovirus*, *Bromoviridae*), apple stem pitting virus (ASPV; *Foveavirus*, *Betaflexiviridae*), tomato ringspot virus (ToRSV; *Nepovirus*, *Secoviridae*), and citrus tristeza virus (CTV; *Closterovirus*, *Closteroviridae*). Indeed, CMV and ToRSV were found widely distributed among EFFs, infecting 38 and 24 species, respectively (Supplementary Table S4). In addition to eudicots, 8 out of 15 virus families also infect monocot species (Figure 4B). Strikingly, members of four families (*Pospiviroidae*, *Avsunviroidae*, *Rhabdoviridae* and *Endornaviridae*) were the only ones infecting all clades of EFFs; it is worth noting that two families (*Pospiviroidae* and *Avsunviroidae*) were constituted by viroids (Figure 4B; Supplementary Table S4; Supplementary Figure S3). Twenty EFFs, for example, were hosts for Hop stunt viroid (HpSVD; *Hostuviroid*, *Pospiviroidae*); most of these plant species belong to Rosales and Sapindales (Supplementary Table S4).

A striking observation is that EFFs species belonging to magnoliids are only infected by a minor number of virus families (Figure 4B). For instance, *P. americana* (avocado) was found to be the host for persea americana alphaendornavirus 1 (PaEV 1; *Alphaendornavirus*, *Endornaviridae*) and two viroids belonging to the families *Pospiviroidae* and *Avsunviroidae*, whereas *A. muricata* (soursop) is only infected by soursop yellow blotch virus (*Rhabdoviridae*) (Supplementary Table S4). Although the reasons behind this interesting observation are far beyond the scope of this work, the molecular determinants should be addressed. It is likely that viral movement proteins and the differences in plasmodesmata biology among the three plant clades could be implicated in the expansion or narrowing of the virus host range [94,95,96,97]. Since the number of plant species analyzed in the present study is biased in favor of EFF species, the application of our approach to all plant species infected by viruses could offer more insights regarding the molecular determinants of host range expansion or shrinking. It is important to note that those gaps along the EFFs in which virus families seem to be absent (Figure 4B) quite likely represent compatible or incompatible plant-virus interactions with no apparent symptoms and, therefore, have not been addressed yet. A high throughput sequencing approach toward detecting exogenous and endogenous viral elements in the aforementioned hosts could offer a full picture of the viral metagenome for these EFFs [98,99,100,101,102].

Aside from the molecular mechanisms governing the observed virus host range distribution, the number and diversity of virus families infecting EFFs offer a picture regarding the future emergence of novel viruses [26,27,102,103,104,105,106,107]. For example, EFFs such as *V. vinifera*, *Cucumis* sp., *Citrus* sp., *Capsicum* sp., and *Solanum* sp., which showed to be infected by the highest number of virus families (Figure 4), could potentially promote virus encounters that might result in recombination and, thereby generate novel and emerging viral diseases. In this regard, grapevine leafroll disease is an excellent example of a multiple viral infection, as it is caused by the association of up to 11 grapevine leafroll-associated viruses [108]. In fact, an extensive survey of five major grapevine viruses indicated a putative recombination event for the grapevine leafroll-associated virus 3 (GLRaV-3; *Ampelovirus*, *Closteroviridae*) [109]. On the other hand, Hanssen et al. found that PepMV recombinants frequently occur in mixed infections under natural conditions [110], showing that coexistence of viruses is a prerequisite for recombination events. In this matter, banana mild mosaic virus (BanMMV), an unassigned genus within the *Betaflexiviridae* family, is often detected in mixed infection with CMV, banana streak virus (BSV; *Badnavirus*, *Caulimoviridae*), and banana bract mosaic virus (BBrMV; *Potyvirus*, *Potyviridae*) [111,112]. Additionally, Xu and co-workers identified a total of eight viruses and one viroid in a single peach tree [101]. All of these examples mentioned, together with the knowledge that plants are mostly infected by multipartite viruses [113], represent a high potential risk for the emergence of new strains by recombination events. This raises the need for a holistic approach to address the virome of important crops such as EFFs. Such an approach could offer not only an overall perspective regarding the interactome of plant viruses in mixed infections (synergistic or antagonistic) but also valuable information in forecasting recombination events in the future. Thus, the information gathered in this review regarding virus families with a wide host range coverage could be helpful for virologists in general, but mostly for epidemiologists who deal with incidence, distribution, and possible control of viral diseases. In the latter case, the efficiency of virus detection could be improved by taking into account the total number of virus families that could infect a determined EFF species, thereby implementing proper disease control strategies for that species.

## 4. Beyond the Visible Symptoms Caused by Plant Viruses: Biochemical, Cellular, and Physiological Changes

Virus infections can be classified as systemic or local. Local infection refers to confinement of virus within and nearby the site of infection, whereas the systemic infection is progressive and starts from the site of infection and then spreads throughout the whole plant using the host vasculature [114,115]. The infected plant can exhibit a variety of symptoms, highly characteristic ones being the induction of colorful patterns (mosaic, chlorosis, necrosis, local necrotic, or chlorotic spots) and distortions (twisting, curling) on leaves, as well as malformation on the branches and discoloration and streaks on the flower petals [60]. In addition, viruses can affect the size, shape, and quality of the fruit, displaying visible symptoms on the fruit such as ringspots, pits, mottling, or line patterns that diminish its appearance and/or organoleptic properties [116]. It is worth mentioning, however, that even though the same virus can infect different plant species, the symptomatology and severity will not be the same [117,118,119,120,121,122,123,124]. This is because the appearance or severity of symptoms depends on the type of virus, the cultivar of the plant, and the host–virus combination [125]. Other factors that can mask or trigger the appearance of symptoms are changes in temperature, light, and/or plant nutrition [126].

Among the common viral symptoms, leaf chlorosis is the consequence of an altered production of pigments within the chloroplasts [127,128,129]. Accordingly, a plethora of changes has been observed in these organelles, such as decrease or clustering of chloroplasts, swelling or atypical shape (amoeboid or globular), formation of vesicles at the periphery, rupture of the envelope, changes in the chloroplast content, disappearance of stroma or dilation of the thylakoid, and disorganized grana, among others [130]. In the case of viral influence on chlorophyll, chlorophyllase was identified as responsible for the emergence of chlorotic spots, ringspots, and mosaic in cucumber [131]. Such a decrease in chlorophyll content was the consequence of increased enzymatic activity of chlorophyllase during infection with CMV. This has also been observed in pumpkin (*Cucurbita pepo* cv Eskandarani) leaves infected with zucchini yellow mosaic virus (ZYMV; *Potyvirus*, *Potyviridae*) [60,125,130,132]. Another symptom related to pigment loss is variegation or breaking, in which flower petals show specks, lines, or sections of tissue with different colorations [60]. Yellowing in leaves, for example, is a characteristic symptom caused by tomato yellow leaf curl virus (TYLCV; *Begomovirus*, *Geminiviridae*) or CTV in tomato and citrus plants, respectively [133,134,135], whereas irregular light/dark green patterns are common symptoms of apple mosaic virus (ApMV; *Ilavirus*, *Bromoviridae*) in apple plants [136,137]. Viral factors such as the coat protein (CP) have been identified as triggering symptoms by interfering with chloroplast development [138,139,140,141,142]. For example, binding of the tobacco mosaic virus (TMV; *Tobamovirus*, *Virgaviridae*) CP to Ferredoxin I causes chlorosis and mosaic in leaves [142,143], whereas the association of the Potato virus Y (PVY; *Potyvirus*, *Potyviridae*) CP with subunits of the RuBisCo enzyme (RbCL) promotes mosaic and chlorosis [144]. Altogether, the viral influence on chloroplasts usually leads to a decrease in photosynthetic activity. A diminished photosynthetic activity during viral infection is the result of a decrease in the synthesis of chloroplast proteins involved in the electron transport chain and in the Calvin–Benson cycle, reducing photosynthesis by up to 50% [125,132,145,146,147,148].

Decrease in photosynthetic activity triggers issued in the development, growth, and/or reproduction of infected plants. A clear example of this is the induced dwarfism in leaves and stems, as well as little or no fruit production, caused by the Banana bunchy top virus (BBTV; *Babuvirus*, *Nanoviridae*) in banana trees [149,150]. However, it is worth mentioning that even though the decrease in plant vigor, yield, and fruit quality caused by virus infections has been attributed to reduced photosynthesis, the causes of this physiological effect are not yet well clarified and should be addressed [125,145,151,152,153]. On the other hand, at the physiological level, viruses interfere in the synthesis, accumulation, storage, and distribution of carbohydrates, which can cause a lack of maturity or flavor in fruits [118]. This is well exemplified in the cases of Cabernet Franc and Cabernet Sauvignon cultivars (*V. vinifera*) infected with grapevine leafroll-associated virus 2 (GLRaV-2; *Closterovirus*, *Closteroviridae*) and grapevine rupestris stem pitting-associated virus (GRSPaV; *Foveavirus*, *Betaflexiviridae*), in which grapes have low taste quality due to poor ripening [118,154,155]. On the other hand, infection of Merlot grape plants with GLRaV-3 caused an accumulation of soluble sugars, leading to feedback inhibition of photosynthesis [153]. Similarly, in the phloem sap of CMV-infected melon (*Cucumis melo*) leaves, high sucrose concentrations and low starch levels were detected, which caused an increase in plant respiration and low photosynthetic rate [156]. Other compounds that contribute to the taste and quality of fruits are organic acids, which decrease by 10% when plants are infected with CTV [157]. Thus, juice volume and ascorbic acid content are considered parameters for establishing the quality of citrus fruits in the industry. In the case of melon plants infected by squash vein yellowing virus (SqVYV; *Ipomovirus*, *Potyviridae*), the content of malic acid increased in fruits, along with changes in the concentration of minerals such as Mg, B, Zn, and K [158]. Some studies have also shown that virus infections increase the protein content of plants, specifically in leaves. For example, Doria et al. [159] used a proteomic approach in *Citrus sinensis* infected with CTV, finding that at least 33 proteins were increased, whereas seven decreased due to infection. Proteins such as superoxide dismutase (SOD), catalase (CAT), guaiacol peroxidase (GPX), and ascorbate peroxidase (APX) were found at high levels, suggesting that *CTV* induces increased oxidative stress. In addition to SOD, CAT, GPX, and APX, accumulation of amino acids, ascorbate, vitamin E, and phenolic compounds has been found and may contribute to preventing cell death due to oxidative stress caused by CTV [116,160].

Physiologically, virus infections impact plant hormones, which are central regulators of plant growth and development [161]. The two most studied defense pathways are jasmonic acid (JA) and salicylic acid (SA) [162]. For instance, studies by Chivasa et al. [163] detail that exogenous application of SA to TMV-infected tobacco plants reduces virus replication. On the contrary, overexpression of viral proteins reduces the expression of SA-responsive genes in *Arabidopsis thaliana* plants infected with TMV, resulting in the suppression or amelioration of defense signaling pathways, and it favors a systemic infection [164]. The same effect was found by Sade et al. [78], in which tomato plants resistant to TYLCV show higher SA concentration. On the other hand, Huang et al. [165] mention that the application of JA and SA in TYLCV-infected plants can increase virus resistance compared to the application of a single hormone. Other hormones, such as cytokines (CK), can decrease viral concentration, diminishing viral symptoms [166,167]. Thus, how viral infections disturb hormone homeostasis and trigger alterations in the plant remains unknown [125,167]. Such complexity of the role of hormones during viral infection is due to the cross-talk between hormones and sugars such as glucose, sucrose, and fructose [78,168].

In summary, the severity of viral diseases depends on several factors, including the type of virus, the cultivar, as well as the host-virus–interaction and environmental conditions. The emerging field of systems biology is expected to reveal not only host components that are important for the virus life cycle but also general patterns about the way in which different viruses manipulate host processes for their own benefit and possible mechanisms by which viruses evade host defenses [97,125,169,170,171,172,173]. Full comprehension of mechanisms underlying events of the plant-virus interaction will be crucial to devise novel plant resistance strategies.

## 5. EFF Yield Losses Caused by Viral Diseases

Agriculture is a very important activity since it provides the primary food supply of more than 7.6 billion people in the world. Its impact is not only restricted to economic and social levels, but agriculture also has a role in mitigating climate change. At the social level, agriculture generates employment worldwide. For example, 34.6% of the population in the world depends economically on agriculture [45]. In terms of economy, the value of global agricultural production in 2019 was estimated at USD 3813 billion, from which EFFs contribute USD 617 billion (16%) [45]. In fact, worldwide production of fruits has increased in the last 5 years, reaching 883 million tons in 2019, which means an increase of 1.4% per year on average [45].

As stated before, the genetic potential of crops yield is not achieved due to abiotic and biotic factors [23,53,54,55]. In the case of viral diseases, they are responsible for important losses, which in terms of economic impact has been estimated at more than USD 30 billion per year [25,26,174,175]. Such impact is scaling rapidly due to agriculture practices (monoculture), changes in vector populations caused by global warming, and direct human intervention in virus spread [25,27,175,176,177,178,179,180,181]. In that sense, nearly half of emerging and re-emerging plant diseases are caused by viruses, forecasting an amplified global economic impact in the near future due to altered temperature and weather patterns. In any case, when viral diseases occur in staple food crops, they are capable of threatening food security, causing famine [182,183,184,185,186].

Although the severity of individual viral diseases may vary with the locality and the EFF variety and from one season to the next [187], the truth is viral diseases represent a major issue in EFF production. Therefore, we expose below the economic losses of some EFFs that have a prominent economic role in the global market (Table 2; Supplementary Tables S2–S4).

### 5.1. Tomato

The scientific name of tomato is *S. lycopersicum*; it is a herbaceous species native to America. Tomato is the most consumed fruit in the world as a vegetable (Table 2; Figure 2B), and it is used to generate multiple products such as paste, soup, juices, and tomato concentrates [188]. The main producing countries of tomato are China, India, Turkey, United States, Egypt, Italy, Iran, Spain, Mexico, and Brazil [45], with yields of around 59.1 t/ha [45].

This plant species is the host for viruses belonging to 15 genera and 12 families (Figure 4A; Supplementary Table S4). Some of them, such as TYLCV, tomato leaf curl virus (ToLCV; *Begomovirus*, *Geminiviridae*), tomato bushy stunt virus (TBSV; *Tombosvirus*, *Tombusviridae*), and beet curly top virus (BCTV; *Curtovirus*, *Geminiviridae*), have caused significant losses in production yields from 18 to 100% [187,189,190]. Members of the *Geminiviridae* family are of great concern because several cases of recombination have been reported [191,192,193,194,195]. In the case of TYLCV, a member of the *Geminiviridae* family has been identified in many parts around the world and is considered a serious threat to tomato production [135,137,189]. TYLCV and its recombinants are transmitted by whitefly called *Bemisia tabaci* and attack both field-grown and greenhouse-grown tomato plants, causing short internodes, small leaves with yellowish edges, and upward curving of the leaf that resembles a spoon [135].

The percentage of tomato production losses worldwide is from 50% to 82%, depending on the growth stage at which the viral infection occurs. On the other hand, the estimated economic impact ranges from USD 46 and 75 billion per year [196]. Other important viruses infecting tomatoes are TMV and Pepino mosaic virus (PepMV; *Potexvirus*, *Alphaflexiviridae*), which cause losses of 19–33% and 20–40%, respectively [197,198,199,200]. Tomato leaf curl New Delhi virus (ToLCNDV) is another begomovirus (family *Geminiviridae*) causing losses in the range of 18–99%, mainly in India and other countries around the world [201,202,203,204]. It has spread to countries such as Spain and Italy, affecting the cultivation of cucurbits [202,205,206]. Therefore, the spread of ToLCNDV to other areas can cause heavy losses not only in tomato or cucurbits but also in up to 43 plant species [125,205]. Finally, another potentially dangerous virus for tomato and sweet pepper is the tomato brown rugose fruit virus (TBRFV; *Tobamovirus*, *Virgaviridae*). TBRFV is transmitted mainly by contact between contaminated plants, as well as by contaminated tools. Currently, TBRFV has been reported in North America (Mexico and the United States), Europe (Germany, Italy, Spain, Greece, France, United Kingdom, and Holland), and Asia (Jordan, Palestine, and China). The symptoms caused by TBRFV are not only yellowing and deformation of young leaves but also discoloration and marbling of the fruits [207].

### 5.2. Bananas and Plantains

Bananas and plantains belong to the genus *Musa* [150]. Their presence in the market does not depend on the season and represents the major source of carbohydrates for more than 400 million people around the world. Bananas and plantains are cultivated in more than 125 countries [208], with the Democratic Republic of Congo, Philippines, Peru, Colombia, Myanmar and the Dominican Republic being among the main producing countries [45].

The source of banana plant reproduction is from young banana suckers, which are removed from the old plantations to establish new fields. It is this practice responsible for virus spread [150], with about 20 virus species from five families infecting banana and plantain crops (Figure 4A; Supplementary Table S4). Among these viruses, the most important in economic terms are BBTV, several species of banana streak viruses (*Badnavirus*, *Caulimoviridae*), and BBrMV [150,209]. The most characteristic symptoms of BBTV are noticed on the leaf and consist of a few dark green stripes or spots on the lower part of the leaf blade. Additionally, smaller leaves with a brittle texture are observed, presenting chlorotic edges and rolled upwards. In younger plants, stunting is observed, and they usually do not produce fruits; however, if fruits are produced, they are twisted [150,209]. BBTV can infect both banana and plantain plants, reducing yields by up to 100% and is therefore considered an economically important disease. This virus is transmitted by the aphid *Pentalonia nigronervosa* or by the use of contaminated plants for propagation. The disease caused by BBTV was first identified in the Fiji Islands and then spread to other countries as a result of human movement and trade [208,210,211]. Until now, its presence has been reported in Australia, Africa, Asia (Malaysia and India), and the Pacific Islands (Polynesia), but it is not found in the Americas [137]. In Australia, BBTV was reported in 1913 and caused a great impact on the banana industry (up to 90% of loss). In Africa, there are no precise estimates of losses, but it is estimated to affect 30–95% plant yield [210,212]. Although banana and plantain producers in the Americas are free of the disease, climate change and the spread of contaminated vegetative material could be catastrophic for country producers in this part of the world.

### 5.3. Cucurbitaceae

The most cultivated and consumed cucurbits worldwide are watermelon (*C. lanatus*), melon (*C. melo*), cucumber (*C. sativus*), and squash (*Cucurbita* sp.) [45]. All these EFFs belong to the *Cucurbitaceae* family and are the second most economically important family after *Solanaceae* [124]. World cucurbit production in 2019 was about 152.8 million tons, with a gross production value of USD 84.3 billion. The main cucurbit producing country is China [45]. In developing countries, cucurbit crops are affected by at least 39 viruses from eight families; however, the severity of infection will depend on climatic and agricultural management conditions for the vectors to transmit the virus [198]. Potyviruses cause great economic losses in developing countries, and their transmission by different aphid species is in a non-persistent manner [213,214,215]. Among the viruses infecting cucurbits are the watermelon mosaic virus (WMV; *Potyvirus*, *Potyviridae*), CMV, and cucumber green mottle mosaic virus (CGMMV; *Tobamovirus*, *Virgaviridae*) (Supplementary Table S4). These viruses cause yield losses in watermelon, melon, squash, and cucumber production [216,217]. WMV is responsible for yield and quality losses in watermelon, cucumber, and squash. Rao and Reedy [187] reported yield losses of 18–73% in watermelon, less than 2% in cucumbers, and 9–49% in squash. The symptoms that occur can vary according to the host; usually, WMV causes dark green mosaic along the veins and leaf blade deformation [218,219]. WMV is distributed worldwide in tropical and subtropical zones; in some countries, such as Mexico, Egypt, Bahrain, and Jordan, WMV is considered a pest [137,213,218]. Another virus with a worldwide distribution is CMV, especially affecting melon, cucumber, and squash crops. This virus induces plant growth retardation, mosaic and leaf distortion, fruit discoloration, and deformation. Losses registered in melon by CMV are up to 2.5% in the United States alone [183]; however, Lecoq et al. [220] reported that almost 100% of the late crop showed symptoms derived from CMV in France, causing considerable yield losses. Finally, CGMMV is a virus that has gained economic importance due to its rapid spread from greenhouse crops to open field crops and to different parts of the world. It was first reported in England in 1935 and is currently found in virtually all of Europe, Asia, some African countries, the United States of America, and Australia [216,221]. Rao and Reddy [183] mention that losses in cucumber crops in England due to viruses such as CMV and WMV, including CGMMV, are up to 15%. Symptoms may vary depending on the cucurbit and occur on leaves, stems and fruits. In the case of melon and watermelon, symptoms on leaves are characterized by mottling, mosaic, brown necrotic lesions (watermelon), and if symptoms are severe, leaf whitening and subsequent wilting of the plant occurs. In watermelon and melon fruits, there are deformations of different degrees. Internally, there are sponginess, yellowing, or rotting, and in the specific case of melon, there are mottling and superficial netting [198]. For squash and zucchini, no symptoms are seen on the plant or fruit surface, but internally there is pulp discoloration or necrosis [206]. At the Lea Valley experimental center, for example, CGMMV decreased the yield of cucumber plants by 15% [222], but losses in the field are greater than 50% [223]. On average, yield losses due to CGMMV are estimated at 40% in melon, watermelon, cucumber, and squash; thus, it is important to promote its control and avoid its spread in virus-free regions.

### 5.4. Apples

Apples (*M. domestica*) belong to the *Rosaceae* family and are one of the most traded fruits in the world (Table 2; Figure 2B). The world production in 2019 was around 87.2 million tons, with a gross production value of USD 50.9 billion. The main producers in the world are China, United States, Japan, Italy, and Chile [45]. Twenty viruses have been reported infecting apple (Figure 4A; Supplementary Table S4), including ApMV, apple stem grooving virus (ASGV; *Capillovirus*, *Betaflexiviridae*), apple chlorotic leaf spot virus (ACLSV; *Trichovirus*, *Betaflexiviridae*), and apple scar skin viroid (ASSVd; *Apscaviroid*, *Pospiviroidae*) [224,225,226].

Infection by ApMV initially starts with yellow spots along the main leaf veins. However, as the disease becomes more severe, leaves may drop prematurely and cause a delay in the apple tree growth. If fruits are produced by these infected trees, they are colorless with a sour taste [227]. Studies conducted by Svoboda and Polák [136] showed that symptoms are more pronounced in spring, so they consider that the virus spreads easily in cold climates. ApMV is mainly distributed in Europe, Oceania, America, and some countries in Africa and Asia [137]. Among the apple cultivars, ‘Golden Delicious′, ‘Granny Smith′, and ‘Jonathan′ are the most susceptible, with losses up to 50% [227,228,229]. Moreover, in a study carried out by Kumar et al. [194], it was found that ApMV, ASGV, ASPV, and ASSVd can generate mixed infections in apple trees, complicating the scenario for disease control strategies.

### 5.5. Grapes

*V. vinifera*, the scientific name of grapes, produces delicious fruits consumed fresh or dried (raisins). Grapes are also the main ingredient for wines, jellies, vinegar, oils, or juices [230]. In 2019, grape production reached 77.1 million tons, which means a gross production value of USD 73.9 billion. China, France, United States, Spain, and Chile are the main countries with grapevine producing areas [45]. *V. vinifera* is a host for at least 90 viruses (Figure 4A; Supplementary Table S4), but only a few of them are of economic importance in the world, such as grapevine fanleaf virus (GFLV; *Nepovirus, Secoviridae*) and several species within the *Closteroviridae* family designated as grapevine leafroll-associated viruses (Supplementary Table S4) [108,109,121,122]. GFLV is transmitted by *Xiphinera index*, an ectoparasite that feeds on plant roots and can resist long periods in the soil. Typical symptoms of GFLV appear early in the growing season and include yellow mottling of leaves, fan-like distortion of leaves, double nodes, short and malformed internodes, as well as poor fruit taste quality and reduced shelf life [117,121,155,231]. This virus is mainly distributed in the wine-growing areas of Europe, America, Africa, and Asia [137], causing losses that range from 10% to more than 80% [117,122]. On the other hand, grapevine leafroll-associated viruses can be transmitted by grafting or mealybugs [155,232]. The first symptoms in adult plants appear on mature leaves, always from the base of the canes, moving progressively upwards to the youngest leaves, which are more pronounced at the end of the growing season, whereas young tissues are usually asymptomatic [121]. Altogether, these viruses can cause yield losses of up to 40% [117]. However, losses in cultivars such as Merlot, Chasselas, and Pinot Noir are up 98% in France, whereas in Italy, losses range between 55 and 65% [117,233].

### 5.6. Citrus

Citrus fruits are among the most commercialized fruits in the world due to their organoleptic, nutritional, and functional properties, both fresh and processed. Among citrus fruits are oranges, lemons, grapefruits, tangerines, and mandarins. The world production of citrus fruits was estimated at 138 million tons in 2019, with a production value of USD 47.7 billion. The main countries with citrus-producing areas are China, France, United States, Spain, and Chile [45,116]. The major problem in the production of citrus fruits is CTV, which infects plants of the *Rutaceae* family but mainly the genus *Citrus* (Figure 4B; Supplementary Table S4) [159]. CTV is transmitted by the spread of plants contaminated with the virus or by aphids such as *Toxoptera citricida*, *Toxoptera aurantii*, and *Aphis spiraecola* [133,159,234]. This virus is widely distributed in the world, and symptoms caused by CTV may vary depending on the genotype of the host [137]. In combinations of grafts and rootstocks such as lemon (*C. macrophylla*), sweet orange (*C. sinensis*), grapefruit (*Citrus x paradisi*), or mandarin (*C. reticulata*) grafted onto sour orange (*C. auramtium*), the virus causes yellowing of leaves, rotting of roots, and even death of the plant in two or three years [133,159,235]. On the other hand, citrus plants tolerant to CTV, such as trifoliate orange (*Poncirus trifoliate*), rangpur lime (*C. limonia*), Cleopatra mandarin (*C. reshni*), and rough lemon (*C. jambhiri*), are used as rootstocks to generate resistant hybrids [236]. Despite these efforts, more than half of the world′s cultivated plants are lost due to the virus [237]. For example, since the appearance of CTV in the 20th century, more than 100 million citrus trees have been lost in the following countries: Argentina, Brazil, United States (California and Florida, mainly), Spain, Israel, Venezuela, Cyprus, Cuba, Mexico, the Dominican Republic, and Italy [133,238]. In Spain alone, one of the main producing countries in the world, it has been estimated that 40 million trees have been lost since 1935 [234]. Recent studies indicate that in Brazil, the yield of Halminton orange (*C. sinensis*) was reduced up to 87%. Similarly, it has been reported that the yield of each plant of Kagzi lime (*C. aurantifolia*) decreases up to 12%. Finally, the yield of oranges and other cultivars (Young Frost Lisbon lemons, tangelos, Frost Washington Navel oranges, tangerines, and Valencia oranges) are lost in California in the range of 40–98% [187].

## 6. Diagnosis of Plant Virus Diseases

Viruses can cause huge economic losses by affecting the quality and productivity of various fruit plant crops like banana, apple, grapevine, citrus, and others [92]. Once infected, plants harbor the virus throughout their life, and visible symptoms of viral diseases appear. So far, it is very difficult to prevent the spread of viruses and their vectors into new territories through international trade, mainly given by the exchange of plant materials across borders [239]. Therefore, early diagnosis of viral diseases is a key factor that determines the timely use of protective measures to confine the viruses, thereby preventing yield losses and a decrease in the quality of fruit products.

The first way to detect viral infections was according to the symptoms they produced. Such an approach was based on biological indexing tests, which were time-consuming and unreliable, especially in cases in which the virus infection was latent or plants exhibited virus-like symptoms unrelated to virus etiology [239,240]. Then, virus detection at the microscopic level began in the late 1930s with electron microscopy; however, this technique cannot be applied for large-scale detection due to the dimensions and costs of the equipment, as well as the operating conditions. Subsequently, the application of serological assay like enzyme-linked immunosorbent assay (ELISA) [241] and nucleic acid-based assay for in vitro DNA amplification called polymerase chain reaction (PCR) [242] represented important advances in plant viral diagnosis. With the progress in molecular biology, nucleic acid-based techniques evolved significantly from conventional PCR to reverse transcription PCR (RT-PCR), nested PCR, multiplex PCR, real-time PCR, immunocapture-PCR (IC-PCR), loop-mediated isothermal amplification (LAMP), recombinase polymerase amplification (RPA), rolling circle amplification (RCA), and microarray and next-generation sequencing (NGS) to improve specificity, sensitivity, and multi-sample processing [239].

Extensive reviews and chapters covering diverse diagnostic methods of viruses infecting plants have been described in the literature [243,244,245,246,247]; therefore, we only provide serological-based and nucleic acid-based approaches that have or could be applied for EFF crops.

### 6.1. Serological Assays

#### 6.1.1. ELISA

ELISA is a cost-effective and robust assay for the routine detection of plant viruses, especially those present in crude extracts, due to its simplicity, detection limit 1–10 ng/mL, ability to analyze a large number of samples, easy interpretation, and semi-quantitative results [248,249]. For virus diagnosis, it is based on the detection of viral antigens, mainly the components of viral particles such as coat protein (CP) subunits, with enzyme-labeled antibodies. The amount of virus present is proportional to the amount of enzyme-labeled antibody, and it is detected by a colorimetric reaction with the enzyme substrate [241]. The direct and indirect ELISA are the most used methods for the diagnosis of plant viruses [250]. Compared to the direct antigen-coated ELISA (DAC-ELISA), the double antibody sandwich ELISA (DAS-ELISA) is the most used test as it is more virus-specific. Currently, DAS-ELISA-based diagnostic kits are used in accredited testing laboratories for routine virus indexing and certification programs for most horticultural crops, including EFFs [251]. A further improvement in the ELISA introduced the use of monoclonal antibodies (MAbs), which specifically detect a particular virus [251]. However, the main limitation of ELISA is the good quality and quantity of antibodies. For example, mixed infections are common in crops of grapevines and citrus [252,253], making it impossible to isolate individual viruses and thereby hindering the obtention of specific antibodies from detecting a particular virus [254]. Recombinant proteins, on the other hand, are an alternative approach for immunogen preparation, especially for viruses in low concentrations. The recombinant approach is fast, economical, and overcomes the problems associated with conventional antigen purification [255]. Additionally, the expression plasmids can be stored for long periods of time, and the recombinant viral proteins are uniform, therefore reducing non-specific antibody recognition. These recombinant proteins are used successfully for polyclonal antibodies (PAbs) production against viruses infecting papaya, bananas, and grapes and to develop immunodiagnostics for routine testing [256,257,258]. In this regard, an important advance was made by producing cocktails of PAbs against the recombinant fusion CP of two and three viruses infecting vegetable crops [259]. Dual and triple cocktails of PAbs were generated for DAC-ELISA toward the detection of CMV and papaya ringspot virus (PRSV; *Potyvirus*, *Potyviridae*) or CMV, PRSV, and groundnut bud necrosis virus (GBNV; *Orthotospovirus*, Tospoviridae) simultaneously in *Cucurbitaceae*, *Solanaceae*, and other hosts [259].

#### 6.1.2. Lateral Flow Assay (LFA)

LFA is an ELISA variant that simplified virus identification [260]. LFA can be done anywhere, with a simple device operated by personnel with little or no training, and results are obtained within minutes [261]. This membrane-based assay involves the use of specific MAbs and PAbs in an immunochromatographic format, incorporating antibody-coated gold or latex particles. A lateral-flow strip consists of a sample application pad, a conjugate or reagent pad, a reaction membrane, and an absorbent pad [262]. There are two types of LFA, the double antibody sandwich format and the competitive format [263]. The sandwich format contains a virus-specific antibody that is immobilized on a membrane, as the test line that captures the viral protein, and a detection antibody-specific antibody is deposited in the control line, capturing the unbound conjugated antibodies. The appearance of the test line indicates the presence or absence of the virus in the sample, and the control line is an internal control to ensure proper functionality of the test [262]. In the competitive format, on the other hand, the detection signal correlates negatively to the antigen concentration. Additionally, these tests have been combined with a novel extraction procedure to allow disease diagnosis in the field. The LFA was successfully applied for the onsite rapid detection of CTV infecting citrus, satsuma dwarf virus (SDV; *Sadwavirus*, *Secoviridae*), and plum pox virus (PPV; *Potyvirus*, *Potyviridae*) infecting apricot, plum, and peach [261,264,265]. LFA has also been useful for the diagnosis of CMV infecting pepper, cucumber, and melon crops, tomato spotted wilt virus (TSWV; *Orthotospovirus*, *Tospoviridae*) infecting pepper and tomato plants [266,267], and PepMV infecting tomato and sweet cucumber [268]. Finally, the development of lateral flow strips has allowed the onsite detection of TBRFV, squash mosaic virus (SqMV; *Comovirus*, *Comoviridae*), tobacco etch virus (TEV; *Potyvirus*, *Potyviridae*), TMV, tobacco ringspot virus (TRSV; *Nepovirus*, *Secoviridae*), tobacco streak virus (TSV; *Ilarvirus*, *Bromoviridae*), ToLCNDV, ToRSV, ZYMV, CGMMV, melon necrotic spot virus (MNSV; *Gammacarmovirus*, *Tombusviridae*), melon severe mosaic virus (MSMV; *Orthotospovirus*, *Tospoviridae*), PepMV, pepper mild mottle virus (PMMoV; *Tobamovirus*, *Virgaviridae*), potato virus X (PVX; *Potexvirus*, *Alphaflexiviridae*), and PVY, among others [269].

#### 6.1.3. Dot Immunobinding Assays

Serological assays such as dot immunobinding assay (DIBA) and tissue blot immunoassay (TBIA) also allow simultaneous screening of large numbers of samples [270]. In these assays, the sap from infected plants is blotted on nitrocellulose or nylon membrane, and the virus is detected by enzyme-labeled secondary antibodies or chemiluminescent probes. DIBA is considered to be simple, rapid, and often more sensitive than ELISA-based techniques [271]. DIBA was adapted for detection of CTV and compared with DAS-ELISA and DAS-indirect ELISA; this assay was easier to perform and as sensitive as either ELISA procedure for CTV diagnosis [272]. This technique was also able to detect the infection with ACLSV and ASGV [273]. Moreover, TBIA was useful for the detection of CMV [274,275].

#### 6.1.4. Computer-Assisted Epitope Identification to Improve Antibody Production

New approaches are emerging in order to improve serological diagnosis. Some involve the identification of epitopes in the viral CP by computer simulation or functionally by epitope mapping and its subsequent artificial synthesis [257]. According to the second approach, two putative CP coding regions (p48 and p37) of banana streak MY virus (BSMYV; *Badnavirus, Caulimoviridae*) were identified in silico by comparison with caulimoviruses, retroviruses, and rice tungro bacilliform virus. A purified fusion protein p37 was injected in rabbits as an antigen for raising polyclonal antiserum. The antiserum was successfully used in antigen-coated plate-ELISA for specific detection of BSMYV in banana fields and tissue cultures raised. On the other hand, the antiserum was also utilized in immuno-capture PCR (IC-PCR) for indexing of episomal BSMYV infection [257]. This bioinformatic approach can be useful for the precise location of CP coding sequences that are not available in any virus.

### 6.2. Nucleic Acid-Based Assays

#### 6.2.1. PCR

Molecular diagnostics began in the mid-1980s with the introduction of PCR, and the first PCR method for virus detection was published ten years later [276]. PCR-based diagnostics can be used for the detection of viruses with DNA and RNA genomes. In the case of RNA viruses, RNA is first reverse transcribed into complementary DNA (cDNA) in a process known as reverse transcription (RT), followed by conventional PCR, involving the amplification of target nucleic acid sequences with primers [277]. Primers can be easily designed using the viral sequence information in databases. Another crucial component for successful PCR is the isolation of good quality DNA, free of endogenous polyphenols, polysaccharides, and nucleases. Some crops, such as bananas, contain very high levels of polyphenols and other secondary metabolites that interfere with PCR amplification. Therefore, an efficient method adapted for nucleic acid isolation is important [239].

Many commercial nucleic acid extraction kits (e.g., RNeasy and DNeasy) have replaced complicated and time-consuming conventional nucleic acid extraction protocols [278,279,280]. PCR-based methods allow the precise detection and characterization of plant viruses, as the amplified products can be sequenced directly or cloned into a suitable vector. Besides reverse transcription PCR, there are other diagnostic techniques based on PCR [281]. *Nested PCR* is useful when the virus titer is very low, the target gene is unstable, and it cannot be checked by electrophoresis due to low amplification product. The product from primary PCR amplification is used for second PCR amplification. Several viruses, including prunus necrotic ringspot virus (PNRSV; *Ilarvirus*, *Bromoviridae*), prune dwarf virus (PDV; *Ilarvirus*, *Bromoviridae*), PPV, and CTV, were detected by this technique. *Co-operational PCR* also requires four primers; however, one is external, and three are internal primers instead of two external and two internal primers associated with nested PCR. This technique shows similar sensitivity to nested PCR, detection in real-time, capability of coupling with dot blot hybridization, it can avoid false-positive results shown at nested PCR, and it also can be applied to capillary air thermal cyclers. This technique has been useful to detect cherry leaf roll virus (CLRV; *Nepovirus*, *Secoviridae*) with higher sensitivity than RT-PCR. *Digital PCR* does not require any reference standards for nucleic acid quantification but rather produces an accurate quantification. The PCR sample is divided into thousands of nanodrops, and after amplification, the drops containing the sequence of the target DNA are detected by fluorescence as positive and those without fluorescence as negative. Then, the statistical analysis of the number of nanodrops gives an exact count of the target DNA, and this helps count the viral charge. Reverse transcriptase droplet digital PCR detected PMMoV in the presence of qPCR inhibitors [282]. Finally, *Multiplex PCR*, *Real-time PCR*, and *Immunocapture-PCR* are described in the following sections. Importantly, PCR has been used successfully for the detection of several viruses that infect citrus in India, including CTV, citrus yellow mosaic virus (CYMV; *Badnavirus*, *Caulimoviridae*), and Indian citrus ringspot virus (ICRSV; *Mandarivirus, Alphaflexiviridae*) [283]. PCR-based diagnostics are also available for viruses that infect bananas, such as BBTV and BSV [284,285] as well as for GLRaV-3 infecting grapevines [286].

#### 6.2.2. Multiplex PCR

Multiplex PCR is a technique for simultaneous and sensitive detection of different DNA fragments in one single PCR reaction [287], and it allows to save reagents and time. Most of the fruit plant crops are infected by more than one virus (Figure 4) containing DNA or RNA genomes that can be detected by multiplex PCR [288,289,290]. Multiple detection is achieved by combining multiple pairs of oligonucleotide primers, each designed to amplify the desired target. Multiplexing requires to design primers that do not show self-complementarity and that exhibit very similar annealing temperatures. Then, PCR products are distinguished by their size or fluorescent tag [290,291,292]. For the purpose of detecting pathogenic microorganisms, multiplex PCR can be performed in various modalities [290]. *Reverse transcription-multiplex PCR* can simultaneously detect various target RNA. After reverse transcription of target RNA, cDNAs are simultaneously amplified with a set of specific primers in a single tube by multiplex PCR. In order to quantify viral load, this technique can be performed in real-time mode, known as *reverse transcription-real-time multiplex PCR*. *Real-time multiplex PCR* uses a set of species-specific primers and probe that is labeled with different fluorescent dyes for each target species so that approximately 2–5 species (depending on the experimental conditions) can be detected simultaneously in a single real-time PCR reaction. Compared to real-time single PCR, real-time multiplex PCR shortens the processing time and reduces the use of reagents. Multiplex RT-PCR has been successfully applied for aphid-borne viruses infecting strawberry, such as strawberry crinkle virus (SCV; *Cytorhabdovirus*, *Rhabdoviridae*), strawberry mild yellow edge virus (SMYEV; *Potexvirus*, *Alphaflexiviridae*), strawberry mottle virus (SMoV; unassigned genus, *Secoviridae*), and strawberry vein banding virus (SVBV; *Caulimovirus*, *Caulimoviridae*) [293]. Furthermore, this technique has been used for simultaneous detection of several viroids within the family *Pospiviroidae* and the ASGV infecting citrus plants [291]. Another example of simultaneous detection by multiplex RT-PCR is the case of viruses infecting grapevines, apples, bananas, and pome fruits [294,295,296,297], as well as for the combination of several viruses and greening bacterium infecting citrus plants [298]. One of the major limitations of multiplexing is the long time taken for optimization of the annealing temperature of the multiple primer sets and the decrease in sensitivity of detection. Additionally, different targets can compete with each other in the reaction in such a way that targets in very low amounts will be hindered by those in high abundance.

#### 6.2.3. Real-Time PCR

One of the limitations of PCR and RT-PCR for virus detection is that PCR products require agarose gel staining with fluorescent dyes such as ethidium bromide, SYBR Green, SYBR Gold, SYBR Safe, Eva Green, GelRed, EZ-Vision, among others [299], which is not convenient for high throughput applications. Additionally, the amount of PCR product is not proportional to the amount of target DNA, and contamination due to the opening of tubes can lead to false-positive results. The real-time quantitative PCR assay is a tool for the detection and quantification of plant viruses. Real-time PCR eliminates agarose gel electrophoresis usage and allows to determine the increase in the amount of amplified DNA through a fluorescent signal [300,301]. It requires the use of nonspecific fluorescent dyes (e.g., SYBR green, LUX, etc.) or specific probes labeled with fluorescent dyes (e.g., TaqMan, Molecular beacon, etc.). The SYBR green dye binds nonspecifically to dsDNA molecules; therefore, the fluorescence generated could be due to specific or nonspecific amplicons or primer dimers. In such cases, melting profiles are used to discriminate primer dimers from actual amplification [251]. On the other hand, probes labeled with fluorescence are specific because more than two independent oligonucleotides need to bind to the target to generate the signal. In contrast to the development of antibodies required for serological tests, real-time PCR has been successfully used for the specific and sensitive detection of viruses [251]. Even though real-time PCR requires very expensive equipment, the overall cost for antibody development is much higher. *Reverse transcription-real-time multiplex PCR* allows detection of various target RNA through simultaneous amplification of cDNAs, produced by reverse transcription, with a set of specific primers in a single tube by multiplex PCR, producing quantitative results [281]. On the other hand, *nested real-time reverse transcription PCR* is a simple and sensitive method for the detection of pathogenic microorganisms. This technique involves a previous reverse transcription step to synthesize cDNA and the use of two external and two internal primers that are complementary to the target sequence, which increases sensitivity, providing quantitative results [281]. In summary, the main advantages of real-time PCR assay are the specificity and speed, high throughput testing, detection of low viral titer, and lesser risk of contamination. For instance, real-time RT-PCR has been successfully used for quantitative detection of CTV and citrus yellow vein clearing virus (CYVCV; *Mandarivirus*, *Alphaflexiviridae*) [301,302,303]. Finally, simplex and duplex real-time PCR assays have been developed for the rapid and sensitive detection of GLRaV-3 and grapevine red blotch virus (GRBV; *Grablovirus*, *Geminiviridae*) in grapevines [304].

#### 6.2.4. Immunocapture-PCR (IC-PCR)

In this technique, viral particles are captured with virus-specific antibodies, followed by the release of viral nucleic acid for PCR amplification. This is very convenient for the detection of plant viruses in which inhibitory plant compounds or low viral titer hinder PCR amplification [305,306]. It has been successfully applied for the detection of BSMYV, which otherwise might lead to false positives with conventional PCR since there are integrated virus sequences in the host genome [307]. In addition to the ability to amplify the episomal viral sequences, IC-PCR also has the ability to concentrate virus particles from crude sap, thus making it more sensitive. IC-PCR is used for routine indexing of *BSV* worldwide [307,308]. It has also been useful for PRSV detection in leaf extracts of papaya and various cucurbits up to 1/10,000 dilution [309], and especially IC-PCR has been developed for GLRaV-3 detection in grapevines [310].

#### 6.2.5. Loop-Mediated Isothermal Amplification (LAMP)

The PCR has been the main tool for viral detection; however, because of temperature cycling, it is time-consuming and less useful than isothermal methods. The LAMP technique has been developed for specific, sensitive, and rapid nucleic acid amplification. This assay uses a group of 4–6 special primers, which together with the strand-displacement activity of *Bacillus subtilis*-derived (Bst) NA polymerase, produce amplicons containing loop regions to which further primers bind, allowing amplification to proceed without thermal cycling [311]. The whole process can be carried out in 1 h at 60–65 °C in a heating block or water bath. Additionally, a pair of loop primers may or may not be used in the reaction. This assay is very useful in high throughput reactions with increased sensitivity and reduced amplification time [251]. LAMP products can be detected by conventional agarose gel electrophoresis or visual observation to estimate turbidity or color changes [312]. There are different LAMP methods for pathogenic microorganisms [313]. *Real-time LAMP* is a constant temperature amplification method carried out at 60–65 °C, for which only a simple water bath is required. This technique eliminates reverse transcription steps, as well as PCR instrument cooling time, which shortens the amplification time. Adding a fluorescent DNA intercalating dye into the reaction enables monitoring of a fluorescence amplification curve. Compared to conventional LAMP assays, this method avoids visible error, enables quantitative detection, and is more suitable for multi-sample analysis. *Reverse transcription LAMP* can synthesize cDNA from template RNA and apply LAMP technology to amplify and detect them. As the template is an RNA sample, in addition to the reagents of DNA amplification, reverse transcriptase is added to the reaction mixture. After mixing and incubating at a constant temperature between 60–65 °C, amplification and detection can be carried out in a single step. *Multiplex LAMP* consists in simultaneous detection of multiple target genes in LAMP reaction, which increases diagnosis specificity. In addition, reverse transcription LAMP in a single tube can be coupled to multiplex LAMP for detection. *Electric LAMP* is based on an electronic simulation that provides fast and inexpensive putative tests of LAMP primers on target sequences compatibility. This aids to determine the opportunity of existing primers to detect recently discovered sequence variants. Finally, *in-disc LAMP* is an integrated device composed of micro-reactors embedded onto compact discs for real-time targeted DNA determination. This method requires similar reagents used in conventional LAMP, and it is performed in a micro-reactor placed in a 65 °C oven. During incubation, the disc is cyclically scanned and optically read to obtain quantitative results. LAMP has been developed and standardized for some of the viruses infecting bananas (BSV, CMV, BBTV and BBrMV), citrus (CYMV), grapevines (GLRaV-3), and apples (ASGV) [314,315,316,317,318,319,320,321]. The LAMP primers can be easily designed using software programs available, such as the PrimerExplorer V5 program [322]. This program is based on six regions in the target sequence, located on the right from the 5′ end and named F3, F2, F1, B1, B2, and B3. The program picks four LAMP primers: forward inner primer (FIP), backward inner primer (BIP), F3, and B3 primers. If needed, loop primer forward (LF) and backward (LB) are designed using the primer information file of the selected LAMP primers. The FIP consists of the F2 sequence at its 3′ end and the same sequence of the F1c region at its 5′ end. The BIP consists of the B2 sequence at its 3′ end and the B1c sequence at its 5′ end. Furthermore, the LF is designed using the complementary strand between F1 and F2 regions, while the LB is designed using the complementary strand between B1 and B2. In addition, the program takes into account four key factors: Tm, stability at the end of each primer, GC content, and secondary structures.

#### 6.2.6. Recombinase Polymerase Amplification (RPA)

In this technique, the isothermal amplification of specific DNA targets is achieved by the combination of proteins and enzymes, such as recombinase, single-stranded binding proteins, and the strand-displacing activity of the polymerase. In combination, all these proteins produce amplicons in 10–15 min at a constant low temperature. There is no need for initial denaturation of the dsDNA target. RPA amplicons can be visualized on gel or by fluorescence and/or hybridization [323,324]. Compared to conventional PCR, which takes ~3 h for analysis, RPA analysis can be completed in just 1 h. Even though the cost of RPA reagents is much higher than in conventional PCR, the overall cost of the assay is reduced because PCR requires expensive thermal cyclers and purification of DNA using commercial kits. RPA is superior to other amplification techniques such as LAMP, which requires larger sets of primers, higher incubation temperature, purified DNA template, and longer incubation time [325], as RPA does not require a purified DNA template and can be easily performed using a very small amount of crude sap extract. Because of its simplicity, sensitivity, and quickness, it is an ideal technique for large scale plant virus indexing. Thus, RPA has been useful for BBTV diagnosis [325], as well as several other viruses such as little cherry virus 2 (LChV-2; *Ampelovirus*, *Closteroviridae*), PPV, tomato mottle virus (ToMoV; *Begomovirus*, *Geminiviridae*), and TYLCV, infecting fruit plant crops [323,324,326].

#### 6.2.7. Rolling Circle Amplification (RCA)

RCA is another isothermal amplification method for viral detection in fruit plants. It uses exo-resistant random hexamer primers and the strand displacement activity of Phi29 DNA polymerase [327] to amplify circular nucleic acids. This technique was first applied for papillomaviruses diagnosis [328] and shortly after for geminivirus infections in tomatoes [329]. RCA is a sequence-independent amplification method carried out at isothermal temperature (30 °C). As random hexamers are employed, the prior sequence information of the viral genomes is not required, and it has the potential to amplify novel circular viral genomes. RCA followed by restriction fragment length polymorphism analysis has been used for the diagnosis of geminiviruses, which have small single-stranded circular DNA genomes [330]. However, the RCA product needs to be sequenced for confirmation of viral origin. RCA has been used even to amplify the bigger viral genomes of badnaviruses infecting bananas, which typically amplify the episomal viral genomes [331]. By employing this strategy, novel badnaviruses associated with the leaf streak disease of bananas have been identified, such as banana streak UA virus, banana streak UI virus, banana streak UL virus, banana streak UM virus, and banana streak IM virus (BSUAV, BSUIV, BSULV, BSUMV; *Badnavirus*, *Caulimoviridae*) [332]. Furthermore, random primed RCA has also been employed to identify the shorter banana streak OL virus (BSOLV; *Badnavirus*, *Caulimoviridae*) variants causing the leaf streak disease of bananas in India [333]. RCA, however, has some limitations: the amplification efficiency decreases with the length of the DNA template, and it is not suitable for larger genomes. Additionally, the probability of strand breaks increases with the length of the DNA molecule, resulting in the termination of the RCA. Furthermore, products generated from complex samples have to be analyzed further to exclude non-specific amplification [327]. RCA had primarily been used to detect plant viruses with small genomes (<3 kb) belonging to the families Geminiviridae and Nanoviridae [330,334]. However, by using a mixture of degenerate primers during the RCA, it was possible to detect plant viruses with larger genomes such as the *Badnaviruses*, *BSV* and sugarcane bacilliform virus (*SCBV*; *Badnavirus*, *Caulimoviridae*), and the *Caulimovirus*, cauliflower mosaic virus (CaMV; *Caulimoviridae*) [332].

#### 6.2.8. Microarray

Some fruit plant crops are infected by a large number of viruses, as is the case of grapevine infected with more than 80 viruses (Supplementary Table S4). In such cases, techniques like ELISA and PCR are limited. In that sense, the development and application of DNA microarrays offer a convenient solution. Although microarrays were originally designed for simultaneous analysis of large-scale gene expression based on complementary base-pairing between the fluorescently labeled target sequences and the spotted probes on a solid surface [335,336], now they are used to detect thousands of plant and animal viruses in a single assay [337,338]. Initially, arrays for plant viruses were developed for the detection of viruses infecting a single crop or few families of plant viruses and viroids [339,340,341,342,343]. Moreover, since synthetic oligonucleotide probes provide greater sensitivity in detection, microarrays containing such probes enabled differentiation among different subgroups (or variants) of CMV and six potato viruses [340,344]. Another microarray containing 150 probes detected 49 viruses, including CMV and other viruses infecting non-fruit plants [345]. Additionally, three *Closteroviridae* members, including GLRaV-4, GLRaV-7, and GLRaV-9, were detected for the first time in Chilean grapevines using an oligonucleotide microarray [343]. A large-scale oligonucleotide microarray developed to identify 538 plant viruses detected CMV, TBSV, TSWV, and MNSV [346]. Besides, it was reported the largest published crop-specific macroarray for the detection of 38 of the most prevalent or emergent viruses infecting grapevine [347]. This array contains 1578 virus-specific 60–70-mer oligonucleotide probes. In a survey of 99 grapevines from the United States and Europe, virus infections were detected in 46 selections of *V. vinifera*, *V. labrusca*, and interspecific hybrids. The majority of infected vines was singly infected, while some were mixed-infected with viruses from two or more families. Representatives of the four main virus families including *Betaflexiviridae*, *Closteroviridae*, *Secoviridae*, and *Tymoviridae* were found alone and in combination [347]. The main limitations of this technique are the high cost of spotting, need for labeled nucleotides, need for dust-free rooms, and little flexibility for use in differentiating strains, as well as the time for processing data [348].

#### 6.2.9. Next-Generation Sequencing (NGS)

NGS technologies, developed in 2005, are massively parallel sequencing platforms that have allowed the rapid identification of viruses and viroids. Today, Illumina technology is the most widely used for sequencing. The process begins with DNA fragmentation and incorporation of adapters that contain segments acting as reference points during amplification, sequencing, and analysis [349]. With this technology, thousands of places throughout the genome are sequenced at once via massive parallel sequencing. NGS technology has made possible the direct identification of viruses and discovery of novel viruses in plants without antibodies or prior knowledge of viral sequences [350,351,352,353]. In response to viral infection, the plant produces small interfering RNAs (siRNAs), complementary to the viral genomic sequences that trigger degradation of viral RNAs in a process known as silencing [354]. Deep sequencing of siRNAs isolated from infected samples allows the recovery of either full or partial genomic viral sequences [351]. These methods have been useful to detect and discover viral infections in many horticultural crops [251,355]. NGS, in addition to its applications in resolving the etiology of viral diseases, characterization, and population genetics, has potential in the high-throughput diagnosis of plant viruses in plant crops [352,355]. The siRNA-based NGS parallel sequencing of symptomatic and asymptomatic samples followed by de novo assembly of long reads has the potential to identify the novel uncharacterized RNA, ssDNA, reverse-transcribing dsDNA viruses, and viroids without prior knowledge of the sequence [351,356]. In addition, conserved domains of viral groups have been identified for different genera or families, which enables designing primers targeting the regions that have the potential to identify different variants and new viruses [356,357]. Several methods to enrich the viral/viroid sequences in a total RNA pool [358], as well as algorithms for the identification of virus/viroid specific nucleic acids, have been developed [359]. In a short period of time, analysis of plant samples by NGS and homology-dependent computational algorithms have identified two new viroids and 49 new viruses from 20 known families [359]. More importantly, the development of user-friendly algorithms for handling voluminous NGS data for viral identification is challenging; however, once optimized to analyze a large number of samples, NGS diagnostics can be used as a reliable tool for certification of horticultural plants destined for exportation. Finally, indexing of the mother plant by NGS, which is used for in vitro large-scale multiplication of crops, can avoid vertical propagation of viral diseases that are a major problem in bananas, citrus and passion fruits.

### 6.3. Biosensors

Biosensors are portable diagnostic devices based on antigen–antibody interactions (immunosensors) or nucleic acid hybridization coupled to a physicochemical transducer microsystem [360]. A biosensor is made up of a receptor, a transducer, and a processor, thereby making this technology economic and highly sensitive for immediate viral detection from leaf extracts. Transducers are classified according to the parameters of measurement as optical (detecting changes in light transmission), thermometric (measuring temperature changes), potentiometric (measuring potential at constant current), amperometric (measuring current at constant potential), cyclic voltametric (measuring current at variable potential), magnetic, or piezoelectric (measuring changes in mass) microsystems. These transducers convert the biological signals into electrical signals of intensity directly proportional to the concentration of a specific analyte [361]. Because of their large surface area, high biocompatibility, and high electron transfer potential, the gold nanoparticles (GNPs) are used to immobilize various biomolecules.

The biosensor based on the bioelectric recognition assay (BERA) is an intelligent system for the detection of plant viruses, combining the principle of artificial neural networks and biosensors. For example, BERA biosensors detect the electric response produced by the interaction of a cell culture suspended in a gel matrix with the CGMMV [362]. This response was indirectly generated, taking into consideration the signal produced by antibody recognition of CGMMV CP. A biosensor based on magnetic immunoassay was developed for the detection and quantification of GFLV, which was achieved through a double-antibody sandwich immunofiltration approach [363]. Additionally, an amperometric biosensor was developed for capsicum chlorosis virus (CaCV; *Orthotospovirus*, *Tospoviridae*) diagnosis, which showed ~1000 times more sensitivity than DAC-ELISA [364]. The antigen sample was placed onto the surface of GNP/multiwalled carbon nanotube screen-printed electrodes in order to interact with polyclonal antibodies specific for CaCV and GBNV. The quartz crystal microbalance (QCM) is a sensitive mass-measuring device consisting of a quartz crystal wafer sandwiched between two metal electrodes connected to an external oscillator circuit that records the resonance frequency [365]. Piezoelectric immunosensors based on artificial or natural antibodies and QCM are able to detect TMV and maize chlorotic mottle virus (MCMV; *Machlomovirus*, *Tombusviridae*), and they could be useful to detect viruses infecting fruit plants [366,367].

An optical miniaturized paper-based DNA sensor identified the early infection caused by BBTV. Some DNA biosensors are based on nucleic acid hybridization and QCM detection of DNA molecules [368,369]. In this regard, the nucleic acid-based QCM biosensors developed to detect cymbidium mosaic virus (CymMV; Potexvirus, *Alphaflexiviridae*) and odontoglossum ringspot virus (ORSV; *Tobamovirus*, *Virgaviridae*) infections in orchids are more sensitive than those based on antibody recognition [370]. Compared to antibodies binding to CP in QCM immunosensors, QCM DNA biosensors possess immobilized nucleic acid sequences that bind more efficiently with their complementary virus CP gene sequences [370]. A potentiometric biosensor was used for the detection of DNA sequences from PPV in plant extracts [371]. Additionally, a cyclic voltametric biosensor was able to differentiate sugarcane white leaf phytoplasma and sugarcane mosaic virus (SCMV; *Potyvirus*, *Potyviridae*) infections [372].

## 7. Disease Management

### 7.1. Horticultural Practices

#### 7.1.1. Use of Disease-Free Propagating Materials and Seeds

Pathogen-free planting material and seeds are fundamental for the fruit industry. Disease-free nurseries should be raised by taking propagative materials from disease-free healthy plants that are identified by making tests of various fruit orchards [373,374,375]. This disease-free material has to be identified and preserved for mass multiplication and distribution of healthy saplings for fruit orchards. Fruit plants such as mango, citrus, guava, grapes, pome and stone fruits, banana, pomegranate, and strawberry, among others, are mostly multiplied by budding, grafting, cuttings, suckers, rhizomes, etc. Thus, if the mother plant carries a transmissible disease, it is passed to the offspring by vegetative propagation [375,376]. Such infected nursery plants serve as one of the important reservoirs for the introduction and spread of diseases and new pathogens into disease-free territories. Citrus, pineapple, plum, and peach viruses are readily transmitted through the use of infected planting material, such as scions or rootstocks, and tools used for nursery production [377,378,379]. These diseases can spread especially long distance and internationally by the importation and use of infected bud wood and its propagation in nurseries. For example, the latent infection in banana suckers results in the introduction of banana bunchy top and mosaic viruses in new plantations [380,381]. Therefore, the production of disease-free bud wood and sapling is very important to manage vegetative transmitted diseases. Virus-free bud wood for citrus nursery propagation and certified planting material has been recommended to control the spread of citrus viruses such as tristeza, greening, ring spot, exocortis, mosaic, witches′ broom, etc. [382]. In that sense, the use of disease-free seeds is convenient for managing PRSV, citrus psorosis virus (CPV; *Ophiovirus, Aspiviridae*), and mulberry ringspot virus (MRSV; *Nepovirus*, *Secoviridae*) [383].

#### 7.1.2. Nutrition

Nutrition is an environmental factor affecting plant survival and resistance to diseases, pathogen virulence, and presence of biocontrol agents. Proper nutrition is a preventive measure for fruit plant disease, whereas wrong or excessive nutrient applications can bring problems [384,385,386]. In addition to C, H and O, thirteen mineral nutrients are generally essential for plant growth, development and production of good yield [384,387]. In general, high nitrogen levels increase susceptibility to many diseases, whereas potassium increases resistance, and the role of phosphorus is variable [388]. For instance, the application of various concentrations of NPK results in a difference in the incidence of pineapple mealybug wilt-associated virus 2 (PMWaV-2; *Ampelovirus*, *Closteroviridae*) [389]. Certain TMV isolates can cause blotchy ripening of tomatoes [390]. In a greenhouse experiment with pot cultures in nutrient solutions, a significant inverse correlation between potassium levels and blotchy ripening was found. The total percentage of blotched tomatoes was highest at the lowest potassium concentration and progressively decreased as the potassium concentration was increased [391]. Tomato internal browning, a chlorotic and necrotic ripening disorder of tomatoes, is also caused by a strain of TMV [390]; however, the severity is influenced by certain environmental factors. When plants were exposed to this virus, potassium produced significantly less internal browning than other treatments exposed to the virus, including phosphorous and phosphorous plus potassium. The infection percentage was not different between fertility treatments and control without virus exposure [392].

#### 7.1.3. Intercropping

Intercropping of certain crops helps in reducing the inoculum of the diseases or population of root-knot nematodes in the fruit crops. These crops produce chemical compounds that inhibit the microorganism population, protecting the main crop from disease. The antagonistic plants release toxic substances in soil that help in reducing the population of plant-parasitic nematodes [393]. The percentage of zucchini plants showing virus symptoms caused by *PRV* was significantly lower in dicultures of zucchini and buckwheat, white clover, okra, or sunn hemp than zucchini monoculture during three years [394]. In contrast, cucurbits as intercrop of bananas should be avoided in order to avoid mosaic disease caused by CMV [395]. Additionally, it has been observed that susceptible weed flora and inter-cultivation of cucurbits should be stopped to reduce the incidence of papaya mosaic virus (PapMV; *Potexvirus*, *Alphaflexiviridae*) [396,397].

#### 7.1.4. Nucellar Embryony

Nucellar embryony is a form of seed reproduction that occurs in certain plant species, such as citrus trees. Nucellar embryos are produced asexually from somatic cells of seed parents [398]. Since citrus viruses are normally restricted to the vascular tissues, virus particles are eliminated in the seedling offspring because there is no direct vascular link between the parent and the nucellar embryos [399,400,401]. Thus, nucellar embryony is an important method for controlling viral diseases. Nevertheless, nucellar seedlings have certain limitations, e.g., they have more thorns, bear late and produce poor quality fruits, but these limitations can be overcome by using apical parts or bud wood from old nuclear limes [402]. Additionally, recovery of mango somatic embryos, particularly in monoembryonic cultivars, eliminates viral pathogens and avoids catastrophic losses frequently occurring in clonally propagated cultivars [403,404]. Finally, advances in nucellar embryogenesis and in vitro culture of apple seed parts could be also helpful in the control of viral diseases affecting apple orchards [405].

#### 7.1.5. Orchard Roguing

Orchard roguing is another important strategy for controlling viral diseases in fruit crops. This method is a cost-effective and environmentally safe disease management practice to identify and eradicate infected fruit plant sources in order to avoid the spread of the disease [406,407]. Once any tree is infected with a virus and viroids, there is no convenient method to eliminate them. The only remedy is to uproot and destroy the infected plants and replant them with certified healthy plants. The removal and destruction of unproductive trees infected by citrus viruses (tristeza, greening, exocortis, ring spot, mosaic, and psorosis, among others) and replanting with certified virus-free planting materials or tolerant rootstocks have worked very well to control viral spread [381,408]. Additionally, other viral diseases of banana, grapes, papaya, pome and stone fruits can be controlled by removing the source of infection and replanting with healthy nursery plants [409,410,411,412]. Banana crops infected with BBTV must be removed from the field to avoid further spread by aphids [413]. Remarkably, the eradication campaign of BBTV had been successful in controlling the disease. In the case of herbaceous crops, such as tomato and pepper, it is also important to maintain good sanitation throughout production and handling [414,415]. This includes the use of clean water, personnel cleanliness, animal exclusion, removing rotten fruit from the fields, cleaning all bins and work surfaces at the end of the day, and maintaining low storage and transport temperatures. An extra benefit of good sanitation to growers and shippers is that it retards infection and reduces decay during shipping and storage.

#### 7.1.6. Destruction and Avoidance of Reservoir Plants

Wild plants and weeds may also serve as reservoirs of both virus and vector [416]. Several crops are short-lived and absent from the field during dry summers, winters or crop rotations. At these times, wild plants frequently harbor viral infections and serve as alternate hosts instead of their usual perennial crops. The destruction of these hosts helps to eliminate the inoculum source. Meyor lemon and *Evodia hupehensis* are symptomless carriers of CTV, and they should be eradicated from the orchards as they act as *foci* for the secondary spread of the virus [417,418,419]. Additionally, elimination of hosts for cherry rasp leaf virus (CRLV; genus *Cheravirus*, family *Secoviridae*), such as balsamroot, dandelion, and plantain, reduces the virus incidence [420]. For example, CRLV and peach rosette mosaic virus (PRMV; *Nepovirus*, *Secoviridae*) are seed-borne of *Chenopodium quinoa*, and their removal helps in controlling the disease [421]. Aside from harboring crop viruses and other pathogens, wild plants act as important reservoirs of insects, mites, and nematodes. Avoidance in the cultivation of tobacco, tomato, cape berry, Zinnia, and various weeds near papaya crops helps in reducing the spread of PLCV by its vector, the whitefly *B. tabaci*. In addition, the cultivation of papaya near cucurbits should be avoided in order to reduce the incidence of PRSV [389]. Therefore, it is highly recommended that the papaya nursery should be raised in an isolated place free from whiteflies.

### 7.2. Vector Control

Insect vectors are the main pathway for the dispersal of plant viruses. The injuries caused by insects at the time of virus transmission may also serve as entry points for the penetration of other pathogens. In this regard, the successful control of these diseases relies on the proper management of insect vectors. The BBTV could be reduced by eliminating the aphid *Pentalonia nigronervosa* with insecticidal sprays, dust, injection, or encapsulation in situ [422]. Additionally, the application of insecticides supplemented with weedicide (2,4-D) is highly effective in killing the aphid and diseased plants [423]. Similarly, the incidence and spread of CTV and PPV can be reduced by controlling the population of their aphid vectors [424,425]. Cherry mottle leaf virus (CMLV; *Trichovirus*, *Betaflexiviridae*) and peach mosaic virus (PcMV; *Trichovirus*, *Betaflexiviridae*) are transmitted by bud mite *Eriophyes inequalis*, and control of the mite reduces the disease incidence [426]. In the case of CRLV and PRMV, which are transmitted by a nematode, the use of fumigants for the control of nematodes helps in reducing the viruses in the orchard [427,428]. On the other hand, the incidence of PRSV can be reduced by planting new plants at least 375 feet away from the main orchard, using physical barriers to avoid contact between winged aphids and papaya seedlings [429]. In addition, papaya crops should be grown in an isolated area with limited cucurbits or old papaya trees, so that plants are subjected to low viral load. Red lady variety of papaya can be successfully grown under protected conditions, and plants remain completely free from papaya leaf curl virus (PaLCuV; *Begomovirus*, *Geminiviridae*) due to protection from whitefly transmission. On the other hand, the spray of fosmite for whitefly control was effective in reducing the disease incidence [430]. Spraying suitable insecticides for the control of mealy bug vectors and ants could reduce the incidence of PMWaV-2 and little cherry virus 1, 2 and 3 (LChV-1, -2, -3; *Closteroviridae*) [431]. Finally, sticky yellow polyethylene sheets are used around the main crop for attracting and sticking to airborne vectors such as aphids and whiteflies. It helps in reducing the incoming population of vectors, as well as disease inoculum reaching the main crop [430].

### 7.3. Thermotherapy

Heat treatments such as sterilization and solarization have been used for reducing or eliminating the inoculum in propagative plant parts, their products and in the soil [432,433]. Soil can be sterilized in a container by passing steam under pressure. Most pathogens are usually killed at a temperature between 60 to 72 °C [434]. Thermo therapeutic treatments are given through steam under pressure, hot water, hot air, and moist hot air. For instance, ASGV and ACLSV can be controlled by thermo therapeutic treatment at 37 °C for four weeks or more [435]. Exposure of infected bud wood at 50 °C for 10 min helps in controlling American plum line pattern virus (APLPV; *Ilarvirus*, *Bromoviridae*) [436]. Heat therapy followed by the isolation and in vitro culture of apical meristems is a suitable procedure to produce virus-free plants. Heat therapy of in vitro shoots at 25–40 °C, increasing 1 °C per day for 18 days, with posterior isolation and culture of apical meristems produced apple and pear plants free from ApMV [437]. Banana mosaic disease caused by BSV can be controlled by exposing infected suckers to heat therapy at 38–40°C for 14 days prior to meristem culture [438]. Additionally, dry heat treatment at 40 °C for one day was effective in curing the infected suckers [439]. Water bath and moist hot air exposure at 50 °C for 120 min contributed to eliminating the ICRSV from the Kinnow mandarin infected buds [440]. Finally, the pineapple wilt virus could be eradicated from infected planting material by treatment with hot water (50 °C for one hour) or dry heat (55 °C for one hour) treatments [441].

### 7.4. Biological Control

Biological control involves the usage of microorganisms for the control of harmful pathogens causing plant diseases without disturbing the ecological balance [442,443]. Usually, parasites and predators are used as biocontrol agents to cope with vectors transmitting viruses and can reduce or eliminate the viral disease [444,445]. Releases of the red-lipped green lacewing *Chrysoperla rufilabris* in caged watermelon contributed to a decrease in the populations of *Bemisia tabaci*, the vector silverleaf whitefly of geminiviruses, in Texas [446]. The greenhouse whitefly *Trialeurodes vaporariorum* is the vector of criniviruses (genus *Crinivirus*) such as strawberry pallidosis associated virus (SPaV; *Crinivirus, Closteroviridae*), tomato chlorosis virus (ToCV *Crinivirus, Closteroviridae*), and tomato infectious chlorosis virus (TICV; *Crinivirus, Closteroviridae*), among others [447]. A biocontrol study showed that the predatory mites *Amblydromalus limonicus* and *Amblyseius swirskii* significantly reduced greenhouse whiteflies densities on greenhouse-grown strawberries [448]. On the other hand, cross protection, in which plants are deliberately infected with a mild strain of a virus, serves as a pre-immunization against a more severe strain of the same virus [449]. Cross protection has been successfully used for the control of CTV, PRSV, and BBTV. In the case of CTV, the virus was controlled using this approach on Pera sweet orange in Brazil, grapefruit in Australia, as well as in grapefruit and sweet orange in South Africa and Japan [450]. In India, acid limes are cross-protected against the severe strain of CTV and gave more yield than uninoculated control [451,452,453]. In Northern India, the existence of mild strain might be giving cross protection against severe and devastating strains. Moreover, the Citrus Improvement Program in South Africa supplies to the growers the high-quality cross protected trees of grapefruit and sweet orange under bud wood certification, thereby avoiding devastation to the citrus industry [454]. Likely, the mild strain of BBTV is widespread in Fiji and has probably been the greatest salvation of banana industry [455].

### 7.5. Chemical Control

The application of chemicals is still the principal method for controlling various plant diseases. An ideal pesticide should be foolproof, highly toxic to pathogens at lower concentrations but safer for human beings or animals. In addition, these chemicals should be cheap, easily available, persistent, easy to spread, easy to handle and apply, stable, and simple to prepare [456]. The chemicals used in crop protection against viral infections are called viricides. Some viricides are currently on the market for PRSV treatment or prevention, such as Virus Stop (Fagro S.A., Mexico), Q 2000 VI (Quimcasa, Mexico), Antivirus (Fertinosa, Mexico) and Ekologik (Bioaga Cellular Biology Lab, USA) [457]. Additionally, a new viricide called Inhibitovir can prevent and reduce disease caused by *PRSV*, comparable to Q-2000VI, with satisfactory protection until harvest [457]. On the other hand, the common broad-spectrum disinfectants Lactoferrin, Virocid, Clorox, and Virkon showed activities against the tobamoviruses TBRFV and CGMMV, with an efficacy of 90–100% [458]. In addition, SP2700 generated a significant effect against CGMMV but poorly against TBRFV. Furthremore, four different chemical-based treatments were able to eradicate ToBRFV from tomato seeds without affecting the ability to germinate [459]: 10% trisodium phosphate solution for 180 min, 4% hydrogen peroxide for 30 min, 2% hydrochloric acid +1.5% sodium hypochlorite for 24 h, and 2.5% sodium hypochlorite solution for 15 min. Additionally, effective treatments for reducing the concentration of ToMV, TMV, and CMV infecting tomato, pepper, melon, and squash seeds were HCl, heated water (65 °C), and ozone (10 g m−3) [460]. These treatments reduced viral concentration in ranges of 51%, 42%, and 32%, respectively. HCl and ozone did not have a negative effect on seed germination. In addition to viricides, homeopathic drugs such as Thuja and cedron have been used to control the papaya viruses PapMV, PRSV and papaya leaf distortion mosaic virus (PLDMV; *Potyvirus*, *Potyviridae*), and TMV, infecting tomatoes [461]. Thuja is also effective in the control of tomato mosaic virus (ToMV; *Tobamovirus*, *Virgaviridae*) [462]. On the other hand, the sorghum extract was effective in the control of SCMV, and could be useful to control viral infections caused by other potyviruses [461].

### 7.6. Use of Disease Resistant Varieties

The use of resistant varieties is the most effective and safest means of controlling crop diseases. Several diseases resistant varieties have been developed in fruit crops; however, there is a lot to be achieved against many important diseases [463,464]. Any gene from wild or unrelated plant species which confers resistance to the pathogen can be transferred to cultivars through traditional means or by genetic engineering [465,466]. By using traditional methods, trifoliate orange and its hybrids, such as Rough lemon, Rangpur lime, Tangelos, Troyer, Yuma citrange, and Volkameriana, have resistance to tristeza disease [466,467]. On the other hand, through overexpression of PRSV CP gene in transgenic papaya plants have been generated papaya plant lines resistant to the virus [468]. Tolerance to TYLCV infection is also associated with single dominant genes in wild tomato species and was successfully introgressed into cultivated tomato [469,470]. In peach, tolerance to PPV (a potyvirus) was mapped to three *loci* [471]. One of these *loci* included a candidate gene with similarities to the *A. thaliana* RTM-2 gene, which is implicated in the restriction of the systemic movement of other potyviruses [471]. However, functional validation will be required to confirm whether the RTM-2-like gene is indeed responsible for the tolerance. Well-characterized exceptions to the NBS-LRR (nucleotide-binding site leucine-rich repeat) configuration of resistance proteins include the non-NBS-LRR-encoding RTM genes. These genes confer dominant resistance to PPV and other two viruses that do not infect EEF plants [472] and the tomato Tm-1 gene, which encodes a protein with a TIM-barrel-like structure that confers dominant resistance to TMV. The Tm-1 protein interacts directly with the viral replicase, impairing viral genome replication [473]. Additionally, to generate fruit plants resistant to viral infections, viral silencing through RNAi has been useful to repress the expression of genes encoding CP or replicase domain gene from PRV [474,475], CP from PPV [476], CP from CMV, ZYMV, and WMV [477]. eIF4E translation initiation factors are essential to plant proteins allowing most +ssRNA viruses to infect plants [478]. Consequently, they are crucial targets for developing genetic resistance. Although often available from crop wild relatives, eIF4E-based resistance may be engineered through TILLING, CRISPR/Cas9, and RNAi. The strategies have relied on knocking out or down the main eIF4E susceptibility factors to generate resistance to PVY and TEV in *Lycopersicum* and *Capsicum* spp., resistance to ZYMV, MNSV, and cucumber vein yellowing virus (CVYV; *Ipomovirus*, *Potyviridae*) in *Cucumis melo*, and resistance to ZYMV and PRSV in *Cucumis sativus*. However, redundancy among eIF4E genes can restrict the efficient use of knockout alleles in breeding. Similar strategies can be extended to other plant factors required by viruses, such as small GTP binding proteins (AtARL8a/b/c), required for ToMV replication, DNA binding protein phosphatase (AtDBP1), and DEAD-box RNA helicase-like protein (AtRH8/PpDDXL), required for PPV and turnip mosaic virus (TuMV; *Potyvirus*, *Potyviridae*) replication in *A. thaliana* and peach [479].

Finally, the generation of virus-resistant varieties also needs to consider the specific climatic conditions for each growing area in order to increase the yield of crops [480,481].

### 7.7. Quarantine and Legislations

Quarantine is the legal restriction on the introduction, movement, and spread of a new pathogen to the disease-free area. With the purpose of preventing the diseases spread to the new unaffected areas, mandatory measures such as bud wood certification, crop inspection, and establishing orchards in regions unfavorable for pathogen are taken [482,483,484]. The plant quarantine can be defined as “the utilization of knowledge by an authority constituted by law, to prevent the entry or spread of injurious plant pests as a service in the public interest” [483]. Therefore, without quarantine measures, new pathogens, diseases, or weeds enter a new country and can spread to become dangerous to the crops. Many fruit diseases like the bunchy top of banana from Sri Lanka to India have been introduced from one region to other causing serious damage to the fruit industry [485]. However, BBTV dissemination has been controlled by quarantine measures and is the most successful example of fruit virus control by this approach [486,487]. Similarly, the prohibition on the importation of infected ornamental cherries, as well as the use of virus-free planting material, has greatly reduced the spread of LChV-2 in British Columbia [488,489].

## 8. Concluding Remarks and Perspectives

EFFs are among the most valuable agricultural food commodities, and the world seems unable to get enough of them, as is reflected by their fastest growth rates of exportation in recent years. However, fruit trees and herbs pass through a series of growth steps or stages before fruits fully ripen, which not only demand continuous nutrient supply across all their growth but also make them susceptible to be infected by plant viruses during this time. To our knowledge, here we have compiled the world′s most comprehensive list of known edible fruits that fit our definition. Moreover, EFFs have been classified taxonomically according to the major clades within the plant kingdom. Additionally, plant viruses infecting the tiny number of EFFs with significant commercial importance in the global market were addressed according to an evolutionary perspective of plant evolution, finding that EFFs belonging to eudicots were hosts for the major number of plant virus families. In addition, the genome composition of virus species infecting commercially important EFFs was mostly represented by ssRNA molecules, whereas other forms of nucleic acid genomes were represented in minor proportions. Remarkably, the host range for some plant virus families showed a wide distribution such as *Bromoviridae*, *Betaflexiviridae*, *Secoviridae* and *Closteroviridae* families. This wide host range distribution, together with the potential co-existence of several viruses in the same host, could lead to the emergence of novel viruses that can threaten the fruit industry in the future. Further, we also have presented the most important approaches for the diagnosis of plant viruses as they are of pivotal importance for the timely use of preventive and protective measures to confine the virus(es) and prevent yield losses. This is particularly important for farmers in developing countries, in which, besides facing policy-related and structural barriers, losses caused by plant viruses are of considerable concern.

Currently, food security is of great concern because the human population is growing, soil fertility is declining, and global warming is changing the weather patterns. Thus, agriculture in general, including EFFs, has to face challenging times in the near future. For instance, COVID-19 outbreaks among workers have challenged the fruit industry by idling processing plants, disrupting supply chains, and farmers have to cope with unprecedented threats to fulfill the demand of EFFs. However, in the age of artificial intelligence, robots and computer vision, the aforementioned issues could be solved by automation. This is what AgTech companies are trying to address with innovative ideas. Therefore, by combining science and technology with agriculture, indoor farming is gaining adherents around the world. Smart farming is not free of challenges, but it could be the solution for humanity because conventional agriculture contributes significantly to the greenhouse gas emissions that are causing climate change. In the case of plant viruses, indoor farming could avoid the impact of these plant pathogens significantly, increasing the yield of crops for an ever-increasing human population. Finally, current fruit tree domestication attempts must consider not only early fructification and high rates of productivity but also the molecular determinants that could confer resistance to viruses.

## Figures and Tables

**Table 1:** Classification of all EFFs of the world.

Species	Genera	Families	Orders	Clade	%
2119	438	119	38	Eudicots	94.8
74	32	16	7	Monocots	3.3
42	15	5	2	Magnoliids	1.8

**Table 2:** The most-traded EFFs in the world.

Fruit	Global Production (Million Tons per Year)	Market Worth (USD per Year)	References
Tomatoes	186	9.4 billion	[45,46,47]
Bananas	116	14.45 billion	[46,48,49]
Watermelons	104	3.74 billion	[46,50]
Apples	86	7.3 billion	[46,51]
Grapes	79	10.9 billion	[46,51]
Oranges	76	14.2 billion	[46,51]

## Data Availability

Data related to virus species and taxonomy were obtained from [58, 84, 85, 86].

## Supplementary Materials

The following are available online at https://www.mdpi.com/article/10.3390/plants11020203/s1, Figure S1: Hierarchical classification of more than 2000 EFFs, Figure S2: Virus families infecting the 64 commercially important EFFs, Figure S3: Host range of virus families infecting the 64 commercially important EFFs, Table S1: An inventory of 2235 plant species producing EFFs, Table S2: EFFs with a prominent value in the global market, Table S3: Virus species infecting EFFs with a prominent value in the global market, Table S4: Classification of EFFs according to plant divergence times and their viral pathogens.
