# Supplementary material for: Viruses Infecting Trees and Herbs That Produce Edible Fleshy Fruits with a Prominent Value in the Global Market: An Evolutionary Perspective
Source: Plants (Basel). 2022 Jan 13;11(2):203. doi: 10.3390/plants11020203 (PMC8778216; doi:10.3390/plants11020203)
Supplement: Supplementary file 1 [file plants-11-00203-s001.zip › plants-1343172- supplementary figures for xml.pdf]

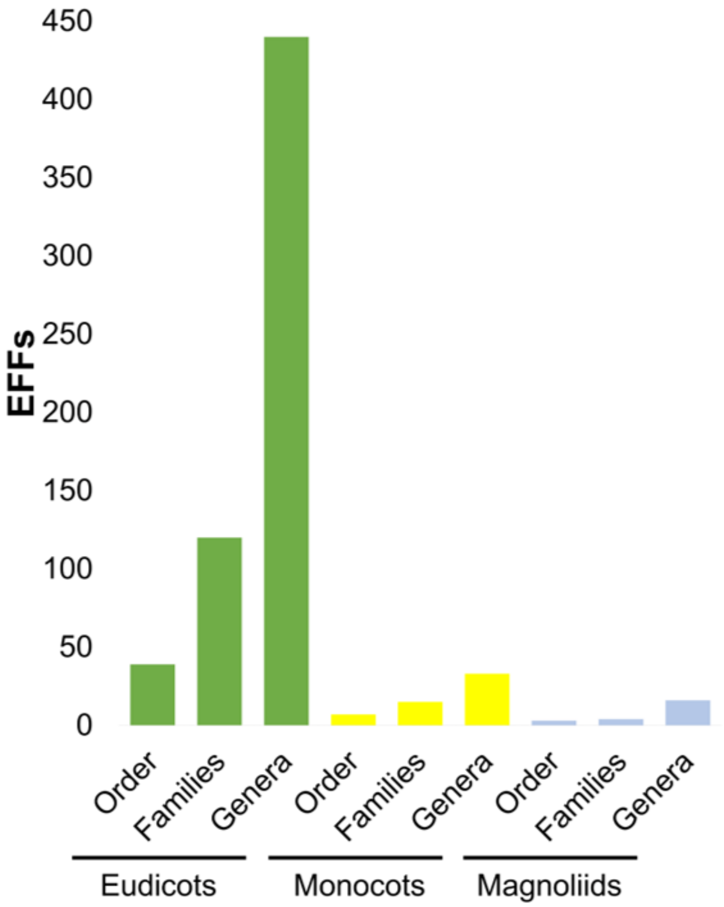

Figure S1. Hierarchical classification of more than 2000 EFFs.

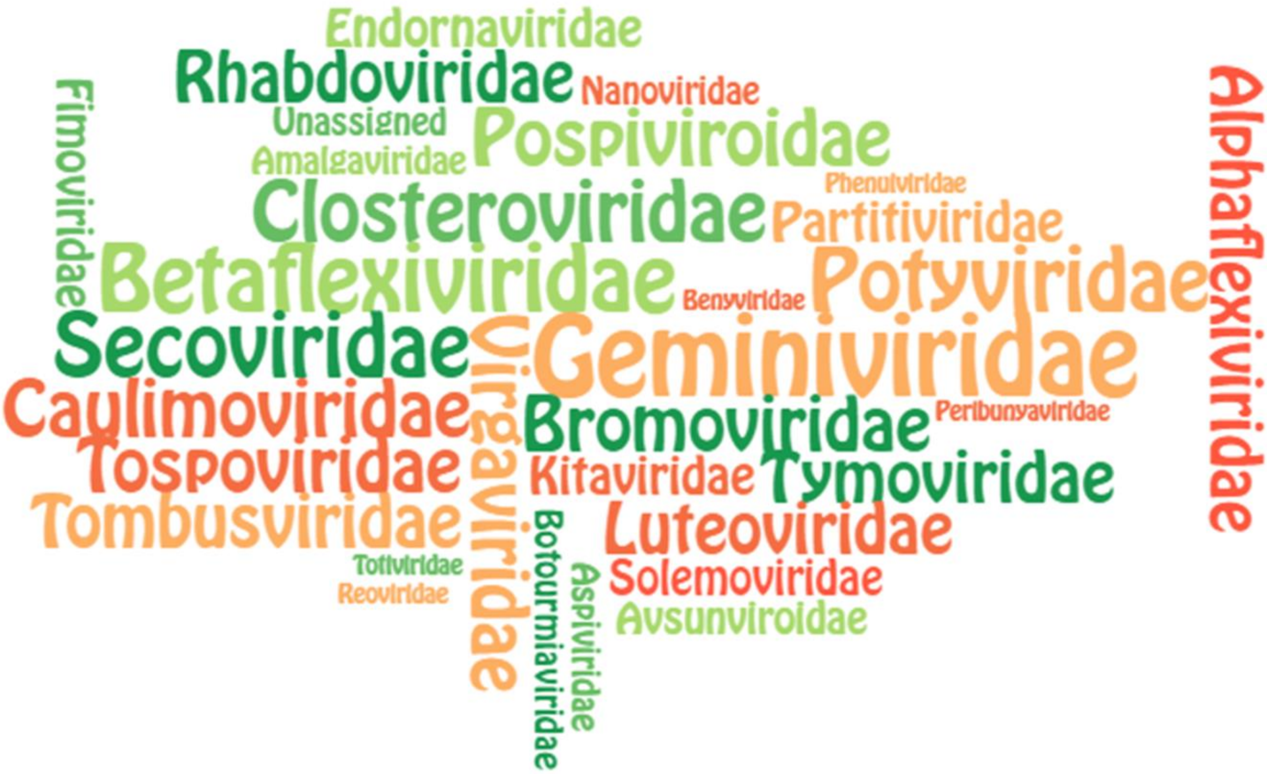

Figure S2. Virus families infecting the 64 commercially important EFFs.

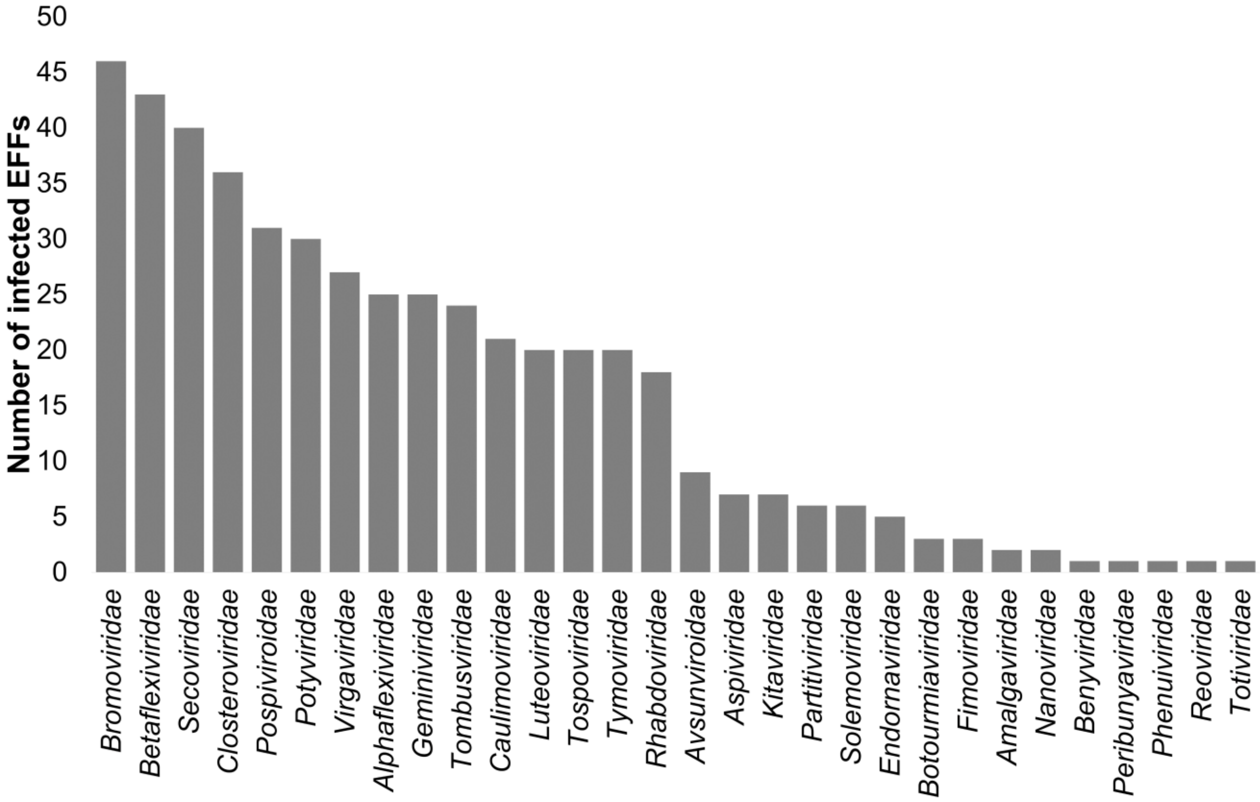

Figure S3. Host range of virus families infecting the 64 commercially important EFFs.
